# Discovery of earth-abundant nitride semiconductors by computational screening and high-pressure synthesis

**DOI:** 10.1038/ncomms11962

**Published:** 2016-06-21

**Authors:** Yoyo Hinuma, Taisuke Hatakeyama, Yu Kumagai, Lee A. Burton, Hikaru Sato, Yoshinori Muraba, Soshi Iimura, Hidenori Hiramatsu, Isao Tanaka, Hideo Hosono, Fumiyasu Oba

**Affiliations:** 1 Department of Materials Science and Engineering, Kyoto University, Yoshida-Honmachi, Sakyo-ku, Kyoto 606-8501, Japan; 2 Center for Materials Research by Information Integration, National Institute for Materials Science, 1-2-1 Sengen, Tsukuba 305-0047, Japan; 3 Laboratory for Materials and Structures, Institute of Innovative Research, Tokyo Institute of Technology, 4259 Nagatsuta, Midori-ku, Yokohama 226-8503, Japan; 4 Materials Research Center for Element Strategy, Tokyo Institute of Technology, 4259 Nagatsuta, Midori-ku, Yokohama 226-8503, Japan

## Abstract

Nitride semiconductors are attractive because they can be environmentally benign, comprised of abundant elements and possess favourable electronic properties. However, those currently commercialized are mostly limited to gallium nitride and its alloys, despite the rich composition space of nitrides. Here we report the screening of ternary zinc nitride semiconductors using first-principles calculations of electronic structure, stability and dopability. This approach identifies as-yet-unreported CaZn_2_N_2_ that has earth-abundant components, smaller carrier effective masses than gallium nitride and a tunable direct bandgap suited for light emission and harvesting. High-pressure synthesis realizes this phase, verifying the predicted crystal structure and band-edge red photoluminescence. In total, we propose 21 promising systems, including Ca_2_ZnN_2_, Ba_2_ZnN_2_ and Zn_2_PN_3_, which have not been reported as semiconductors previously. Given the variety in bandgaps of the identified compounds, the present study expands the potential suitability of nitride semiconductors for a broader range of electronic, optoelectronic and photovoltaic applications.

## Abstract (web summary)

Nitride semiconductors are attractive for various applications. Here a computational screening study identifies a number of possible ternary nitrides as promising candidates, including a phase that the authors synthesize for the first time via a high-pressure route.

## Graphical abstract

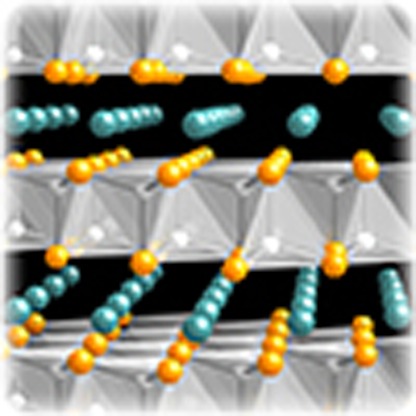

Semiconductors are increasingly relied upon in modern society. Prototypical elemental semiconductors, Si and Ge, play principal roles in electronics and photovoltaics. Compound semiconductors including GaAs, GaP and GaN have not only enhanced these applications, but also paved the way to optoelectronic devices, such as light-emitting diodes^1^. Current electronics also utilize oxide semiconductors in thin-film transistors^2^, and semiconductor-based photocatalysts and photoelectrochemical cells are being developed, particularly for the electrolysis of water^3^,^4^. With such a variety of applications, the exploration of novel semiconducting materials has been a fundamentally and technologically important issue. Among the compound semiconductors, nitrides are attractive due to the abundant and environmentally benign nitrogen constituent. Many nitrides possess relatively high chemical stability at high temperature, which is advantageous for applications under severe environments such as power electronics. Moreover, bulk crystal and film growth techniques are well established, especially for group III nitrides. However, the currently commercialized nitride semiconductors are mostly limited to GaN and its based Al_*x*_Ga_1−*x*_N and In_*x*_Ga_1−*x*_N alloys with tuned bandgaps. This situation has stimulated experimental and computational searches of novel nitrides, for instance, spinel-Si_3_N_4_ (ref. 5), cubic-Zr_3_N_4_ (ref. 6), ZnSnN_2_ (ref. 7), CuNbN_2_ (ref. 8) and LaWN_3_ (ref. 9). In particular, recent developments of high-throughput first-principles screening based on prototype crystal structures and evolutionary algorithm structure searches have enabled the accelerated identification of a variety of previously unreported or uncharacterized semiconductors, including nitrides^8^,^9^,^10^,^11^,^12^,^13^,^14^,^15^,^16^,^17^,^18^,^19^,^20^.

High carrier mobility is desirable or even mandatory, in most applications of semiconductors, and the effective mass is a relevant fundamental physical parameter. Group III nitrides in the wurtzite structure, especially GaN and InN, have small electron effective masses due to spatially diffuse cation *s*-orbital contributions to their conduction bands. Moderate hole effective masses are also attained in the valence bands with mainly N-2*p* characteristics. Technological uses for nitride semiconductors could be expanded if alternative materials with similar desirable electronic properties were found. Ideally, such alternatives should be environmentally benign and comprised of earth-abundant constituents. A chemical analogy to GaN and InN suggests that the nitrides composed of Zn, Ge or Sn would be good candidates. In particular, hybridization between closed-shell Zn-3*d* and N-2*p* states in the valence band is expected to reduce hole effective masses in Zn nitrides. Indeed, a Zn binary nitride, Zn_3_N_2_, possesses such a band structure^21^, but the fabrication of its high-quality film is still challenging, partly due to its small exothermic formation enthalpy (−22.6 kJ mol^−1^ or −47 meV per atom)^22^. In addition, well-known ternary zinc nitride semiconductors are limited to a small number of systems, such as LiZnN (ref. 23), ZnGeN_2_ (ref. 24) and ZnSnN_2_ (ref. 7).

Here we report computational screening of ternary zinc nitrides using a combination of the prototype-based and evolutionary algorithm structure searches. Twenty-one promising semiconductors with small carrier effective masses are identified via systematic first-principles calculations of stability and electronic structure. The proposed nitrides include previously unreported CaZn_2_N_2_ with earth-abundant components and a direct bandgap, which is synthesized by high-pressure methods. Alloy calculations indicate a bandgap capable of covering most of the visible light range. Native defect and dopant calculations also predict *p*- and *n*-type dopability for CaZn_2_N_2_ and the related Ca_2_ZnN_2_.

## Results

### Theoretical identification of ternary zinc nitride semiconductors

Systems of interest have been selected from 583 existing and hypothetical ternary zinc nitrides, which consist of various polymorphs of 125 chemical formulae. These candidate compounds are constructed using 52 prototype crystal structures, either reported in the Inorganic Crystal Structure Database (ICSD)^25^ or previously predicted theoretically^15^ (Supplementary Table 1). The considered structures are taken by N^3−^ compounds, that is, nitrides, involving two kinds of cations, at least one of that is divalent; those taken by azides, diazenides and pernitrides are excluded. The divalent cation is replaced with Zn(II) and the other is replaced with a cation of the same valence (Supplementary Table 2), which is expected to yield a closed-shell electronic structure and therefore exhibit a quantifiable bandgap. The cation species are not restricted to abundant elements at this stage because the electronic structure of compounds involving any element can provide useful information on the design of novel semiconductors. Subsequently, an evolutionary algorithm^12^,^26^ is used to validate or update the most stable crystal structures. This allows us to overcome the limitations associated with relying on already reported crystal structures in the ICSD.

The screening is performed from various aspects: (i) the electronic structure, that is, bandgaps and effective masses; (ii) dynamic stability against lattice vibration; and (iii) thermodynamic stability against competing phases in the phase diagram. Here, not only thermodynamically stable phases, but also metastable phases with small positive formation energies (<50 meV per atom) are selected. This tolerance partly accommodates errors associated with the approximations used in first-principles calculations and the omission of temperature effects (Supplementary Method 1). Although metastable phases with higher formation energies may be grown by the use of high pressure, high temperature and/or non-equilibrium conditions, we prioritize the predictions of thermodynamically stable or slightly metastable compounds for the ease of the fabrication. Furthermore, the energetics of relevant native point defects and dopants is assessed to identify the dopability into *p* type, *n* type or both for promising compounds. Details of the screening and computational procedures are described in the Methods section and Supplementary Method 1.

**Figure 1:**
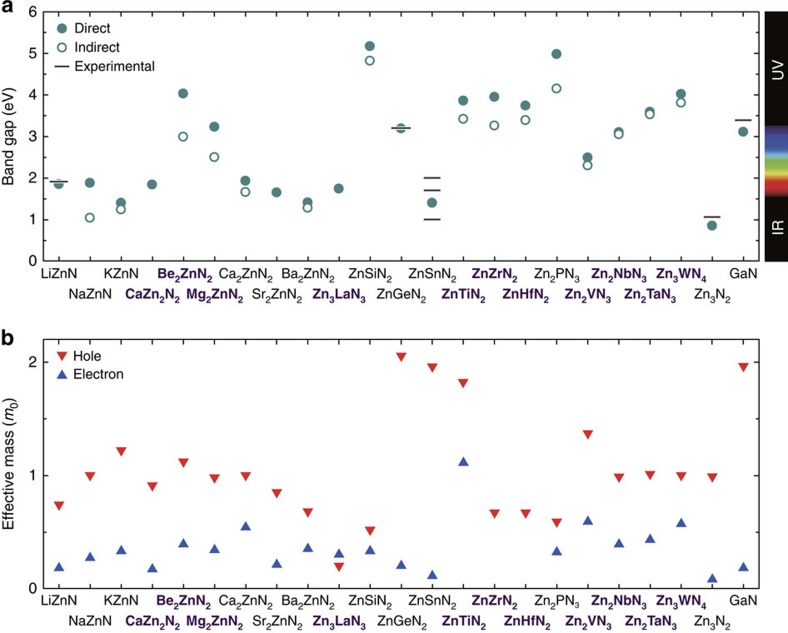
Electronic properties of theoretically identified ternary zinc nitrides. (**a**) Bandgaps. Filled and open circles denote the values for direct and indirect gaps, respectively. Available experimental values are shown with bars (see Supplementary Table 4 for tabulated values and references). (**b**) Effective masses for holes and electrons normalized by the free-electron rest mass, *m*_0_. The values for the directions that provide the smallest effective masses are shown (see Supplementary Table 5 for tabulated values). Heavy holes are considered when the valence band maximum is degenerate or nearly degenerate (<0.1 eV differences in band energies). The values for Zn_3_N_2_ and GaN are also shown as references. As-yet-unreported nitrides are indicated with a bold purple font.

Identified from the computational screening are 21 nitrides that are dynamically stable and thermodynamically stable/slightly metastable against competing phases (with formation energies that are <50 meV per atom), possess bandgaps and exhibit small effective masses for holes, electrons or both (smaller than 2*m*_0_, where *m*_0_ denotes the free-electron rest mass); their crystal structures, phonon densities of states, phase diagrams and electronic band structures are presented in Supplementary Figs 1–4 and Supplementary Table 3. Here, heavy holes are considered when the valence band maximum (VBM) is degenerate or nearly degenerate, as their dominant contributions to the electronic density of states near the VBM mean that heavy holes are most relevant to hole conduction. The bandgaps and effective masses of these nitrides are shown in Fig. 1, alongside those of Zn_3_N_2_ and GaN. Our screening identifies 11 nitrides that have not been reported in the ICSD, Be_2_ZnN_2_, Mg_2_ZnN_2_, CaZn_2_N_2_, Zn_3_LaN_3_, ZnTiN_2_, ZnZrN_2_, ZnHfN_2_, Zn_2_VN_3_, Zn_2_NbN_3_, Zn_2_TaN_3_ and Zn_3_WN_4_. It has also selected 10 already known nitrides, validating our computational methods and screening criteria: LiZnN (ref. 23), Ca_2_ZnN_2_ (ref. 27), Sr_2_ZnN_2_ (ref. 28), Ba_2_ZnN_2_ (ref. 28), ZnSiN_2_ (ref. 24), ZnGeN_2_ (ref. 24), ZnSnN_2_ (ref. 7) and Zn_2_PN_3_ (ref. 29) reported experimentally, and NaZnN and KZnN predicted theoretically^15^. We note that four of these known nitrides, that is, Ca_2_ZnN_2_, Sr_2_ZnN_2_, Ba_2_ZnN_2_ and Zn_2_PN_3_ have not, to our knowledge, been considered as semiconductors previously and their appealing band structures are unveiled in the present study. In addition, the majority of the identified nitrides consist solely of abundant elements.

Among the known nitrides, ZnSiN_2_, ZnGeN_2_ and ZnSnN_2_ are found to favour a wurtzite-derived crystal structure in consistency with previous experiments^7^,^24^. ZnTiN_2_ is predicted to take this crystal structure among the newly identified Zn–IV–N_2_ compounds, whereas ZnZrN_2_ and ZnHfN_2_ are energetically more favourable with an alternate, *P*3*m*1 crystal structure obtained using the evolutionary algorithm. The present results extend the wurtzite derivatives from Zn–IV–N_2_ to those involving group V and VI elements. Zn_2_PN_3_ can be regarded as a prototype of such kind with a group V element. Our calculations suggest that Zn_2_VN_3_, Zn_2_NbN_3_ and Zn_2_TaN_3_ are stable in this Zn_2_PN_3_ structure. In addition, Zn_3_WN_4_ is predicted to take a wurtzite-derived structure as well. In total, 10 unique structure types are observed for the 21 identified nitrides (Supplementary Table 3; Supplementary Fig. 1).

The hole and electron effective masses of many of the identified nitrides are comparable to or even smaller than those of Zn_3_N_2_ and GaN. In particular, much smaller hole effective masses than that of GaN (2.0*m*_0_) are recognized for a number of ternary zinc nitrides. This is partly attributed to the hybridization of the Zn-3*d* orbitals near the VBM and is appealing in view of potential applications utilizing *p*-type conductivity. Especially, Zn_3_LaN_3_ shows a non-degenerate VBM with an exceedingly small hole effective mass of 0.2*m*_0_ (Fig. 1b; Supplementary Fig. 4). Moreover, some of the identified nitrides have direct bandgaps that are advantageous for light emission and harvesting. Indirect-gap compounds with slightly larger direct gaps can also be good candidates for photoabsorbers in solar cells^13^,^15^. Identified nitrides having such indirect or direct band structures and exhibiting good matching with the solar spectrum (bandgaps of ∼0.8 to ∼1.9 eV) include LiZnN, KZnN, CaZn_2_N_2_, Sr_2_ZnN_2_, Ba_2_ZnN_2_, Zn_3_LaN_3_ and ZnSnN_2_ (Fig. 1a). In particular, the present study reveals the properties of Ba_2_ZnN_2_ with a theoretical indirect gap of 1.3 eV and a high absorption coefficient of 5 × 10^4^ cm^−1^ near the absorption threshold at 1.5 eV, which are suited for thin-film photovoltaics (Supplementary Fig. 5). Compounds with rather wide gaps (>4 eV) together with small effective masses are of interest for potential applications in power electronics, where wider gaps allow for higher breakdown voltages. Promising systems in this respect are ZnSiN_2_ and Zn_2_PN_3_.

**Figure 2:**
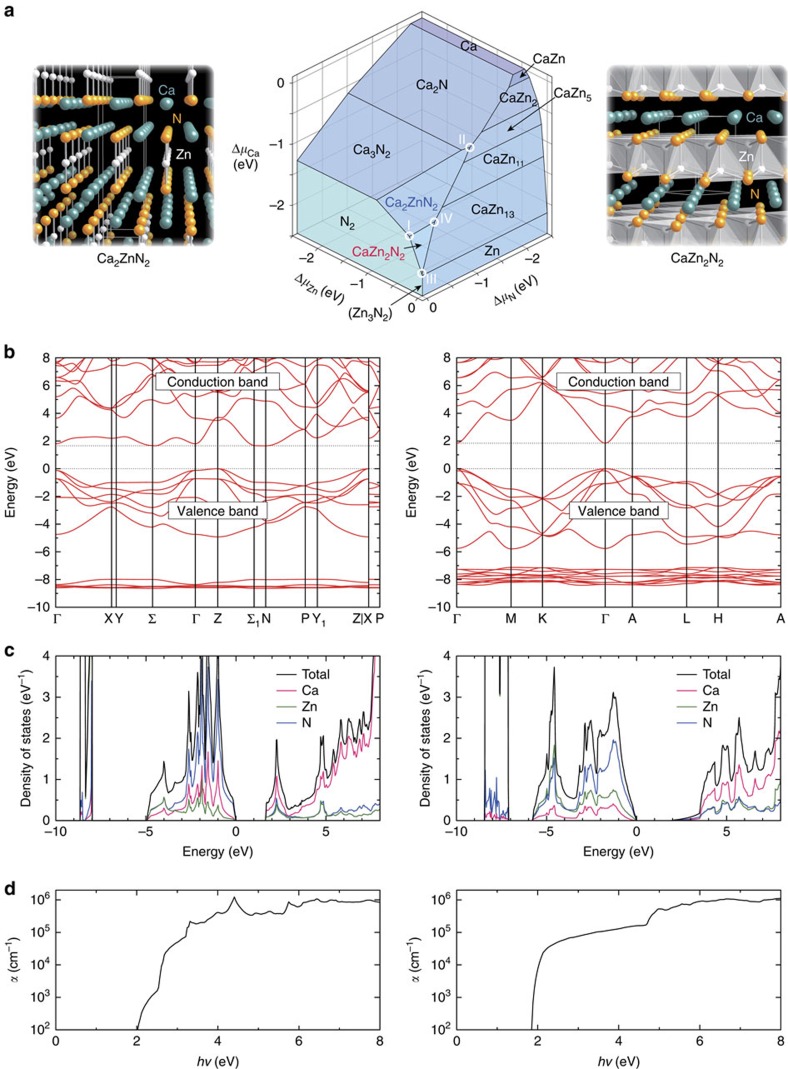
Theoretically predicted characteristics of Ca_2_ZnN_2_ and CaZn_2_N_2_. (**a**) Ca–Zn–N ternary phase diagram at 0 K and 0 GPa, showing the stable region of Ca_2_ZnN_2_ and CaZn_2_N_2_ in the chemical potential space. The chemical potentials Δ*μ*_*i*_ (*i*=Ca, Zn and N) are relative to those at the standard states, which are taken to be the Ca and Zn metals, and the N_2_ molecule. The Zn_3_N_2_ phase is metastable and indicated in parenthesis. Chemical potential conditions I–IV are considered in the discussion of point-defect energetics. On the left and right sides of panel are the crystal structures of Ca_2_ZnN_2_ and CaZn_2_N_2_, respectively. The frames represent the conventional unit cells (*I*4*/mmm* (tetragonal) and 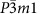 (trigonal), respectively). The Ca atoms (green) and Zn atoms (white) are coordinated by five and two N atoms (yellow), respectively, in Ca_2_ZnN_2_, whereas by six and four N atoms in CaZn_2_N_2_; only Zn–N bonds are illustrated for easy visualization. (**b**) Electronic band structures. (**c**) Total and site-projected electronic densities of states per formula unit. (**d**) Absorption spectra (*α*: absorption coefficient; *hν*: photon energy). For **b**–**d**, the left and right sides of each panel show results for Ca_2_ZnN_2_ and CaZn_2_N_2_, respectively.

Among the identified nitrides, Ca_2_ZnN_2_ and CaZn_2_N_2_ are especially attractive due to their earth-abundant constituents, and small hole and electron effective masses. The synthesis of Ca_2_ZnN_2_ has been reported^27^, but the electronic properties have not thus far been established experimentally. Electronic density of states from a first-principles calculation has been reported in ref. 30 and our result shown in Fig. 2c is similar in the shape of the valence and conduction bands. Ca_2_ZnN_2_ is predicted to have an indirect-type band structure with a minimum bandgap of 1.7 eV (Figs 1a and 2b, left of panel). Ca-3*d* states mainly constitute electronic states near the conduction band minimum (CBM) (Fig. 2c, left of panel); nevertheless, the electron effective mass is as small as 0.5*m*_0_ for a particular direction (Σ–Γ), and a small hole effective mass of 1.0*m*_0_ is also appealing (Fig. 1b). The optical absorption spectrum shows a threshold of ∼2.0 eV, which is ∼0.3 eV above the indirect-gap (Fig. 2d, left of panel).

The other ternary phase, CaZn_2_N_2_, has not been reported previously to the best of our knowledge. Our calculations predict that this phase is stable against the known competing elementary, binary and ternary phases, including Ca_2_ZnN_2_ (Fig. 2a). It takes a relatively simple trigonal structure, where the Ca and Zn atoms are coordinated by six and four N atoms, respectively (Fig. 2a). The highest valence states have an N-2*p* characteristic with sizable contributions of Zn and Ca orbitals (Fig. 2c, right of panel). The cation *s* orbitals mainly constitute electronic states near the CBM in stark contrast to the case of Ca_2_ZnN_2_. These bands show large dispersions (Fig. 2b, right of panel) and thereby yield small effective masses of 0.9*m*_0_ and 0.2*m*_0_ for heavy holes and electrons, respectively (Fig. 1b); the heavy hole effective mass is about half the value of GaN and the electron effective mass is comparable to that of GaN. The symmetrically allowed electronic transitions over the direct gap lead to a steep optical absorption threshold and slightly above that, the absorption coefficient reaches as high as 5 × 10^4^ cm^−1^ (Fig. 2d, right of panel). The direct bandgap of 1.8 eV (Figs 1a and 2b, right of panel) corresponds to a red region in visible light when light emission is considered. It would also be suitable as a solar cell photoabsorber, given the theoretical photovoltaic energy conversion efficiency at the Shockley–Queisser limit is 27% for this bandgap under the air mass 1.5 G sunlight^31^. The bandgap can be narrowed via alloying towards a higher theoretical efficiency, as discussed later.

### Experimental verification of Ca_2_ZnN_2_ and CaZn_2_N_2_

**Figure 3:**
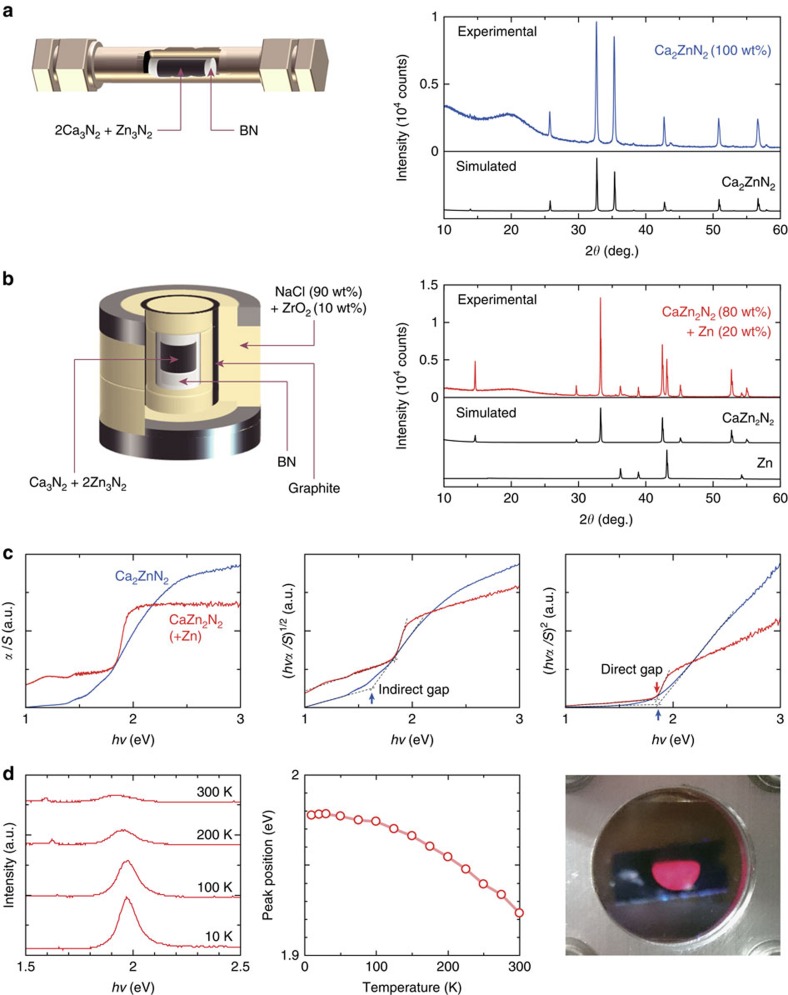
Experimental verification of the Ca_2_ZnN_2_ and CaZn_2_N_2_ phases. Schematics of experimental set-ups and X-ray diffraction profiles for (**a**) a polycrystalline sample with a starting composition of Ca:Zn:N=2:1:2 aiming at the formation of the Ca_2_ZnN_2_ phase, which was encapsulated in a sealed steel tube and annealed at 680 °C for 40 h, and (**b**) a polycrystalline sample treated at 1200 °C and 5.0 GPa for 1 h with a starting composition of 1:2:2 corresponding to CaZn_2_N_2_. Also shown are simulated profiles that were obtained on the basis of the theoretically predicted crystal structure of CaZn_2_N_2_ and the reported crystal structures of Ca_2_ZnN_2_ and Zn. The broad peaks around 2*θ*=20° originate from sample capsules used in the measurements. (**c**) Absorption spectra of Ca_2_ZnN_2_ and CaZn_2_N_2_ derived from diffuse reflectance spectra at 300 K and the Kubelka–Munk relation (*α*: absorption coefficient; *S*: scattering factor; and *hν*: photon energy). (**d**) Photoluminescence (PL) from CaZn_2_N_2_: PL spectra at 10, 100, 200 and 300 K (left of panel), the temperature dependence of the PL peak position (middle of panel) and a photograph of red PL at 10 K (right of panel).

The theoretical phase diagram of the Ca–Zn–N ternary system indicates that the stable region of the newly identified CaZn_2_N_2_ phase is limited to high nitrogen chemical potential conditions. The corresponding nitrogen partial pressure is much higher than 0.1 MPa at, for instance, 800 K and above (Fig. 2a; Supplementary Figs 6 and 7; Supplementary Note 1). Therefore, we have utilized high-pressure synthesis that can allow access to such conditions. Conventional synthesis without applying external pressure successfully forms the previously reported Ca_2_ZnN_2_ (Fig. 3a) and predominantly yields this phase even with the starting composition corresponding to CaZn_2_N_2_ (Supplementary Fig. 8). Using high pressure, trigonal CaZn_2_N_2_ is obtained as the primary component with a minority presence of Zn metal (Fig. 3b; Supplementary Fig. 9; Supplementary Tables 6 and 7); the origin of Zn metal segregation is a chemical reaction between the Ca component and BN in the high-pressure cell. This result demonstrates that high-pressure synthesis is effective to realize complex nitrides that are stable only under high nitrogen partial pressure conditions. The experimentally determined lattice parameters of CaZn_2_N_2_ are *a*=3.46380(11) Å and *c*=6.00969(30) Å. These values are close to theoretically predicted lattice parameters of *a*=3.454 Å and *c*=5.990 Å, with 0.3% differences between experiment and theory for both *a* and *c*. The Ca to Zn ratio measured for a CaZn_2_N_2_ region using the electron probe micro-analyzer is 1.00(1):1.97(3), which is almost exactly the target composition.

As mentioned above, our calculations predict that the previously reported Ca_2_ZnN_2_ is an indirect-type semiconductor, whereas the newly identified CaZn_2_N_2_ is direct-type. Corresponding to such differences in theoretical band structures and absorption spectra between the two phases (Fig. 2b,d), CaZn_2_N_2_ exhibits a steeper absorption threshold, with an energy of ∼1.9 eV at 300 K (Fig. 3c). Furthermore, red photoluminescence originating from a band-to-band transition is observed (Fig. 3d). The band-edge luminescence peak is located at 1.98 eV at 10 K and gradually shifts to lower energies with increasing temperature, down to 1.92 eV at 300 K. It should be noted that the red luminescence is visible even at room temperature (Supplementary Fig. 10) and no deep-level emissions are observed despite the polycrystalline nature. Therefore, this nitride is a promising candidate not only for a photoabsorber, but also a red light emitter. The diffuse reflectance and photoluminescence results indicate that the direct bandgap of CaZn_2_N_2_ is ∼1.9 eV at room temperature. This value is close to the theoretically predicted direct gap of 1.83 eV. The bandgap of the Ca_2_ZnN_2_ phase has not been reported either, and we estimate its indirect and direct gaps to be ∼1.6 and ∼1.9 eV from the diffuse reflectance, respectively, again in good agreement with the corresponding theoretical values of 1.65 and 1.92 eV. The theoretically proposed earth-abundant nitride, CaZn_2_N_2_, is thus verified experimentally, as well as Ca_2_ZnN_2_ whose fundamental properties have not been well established previously.

### Predictions on the bandgap engineering of CaZn_2_N_2_

**Figure 4:**
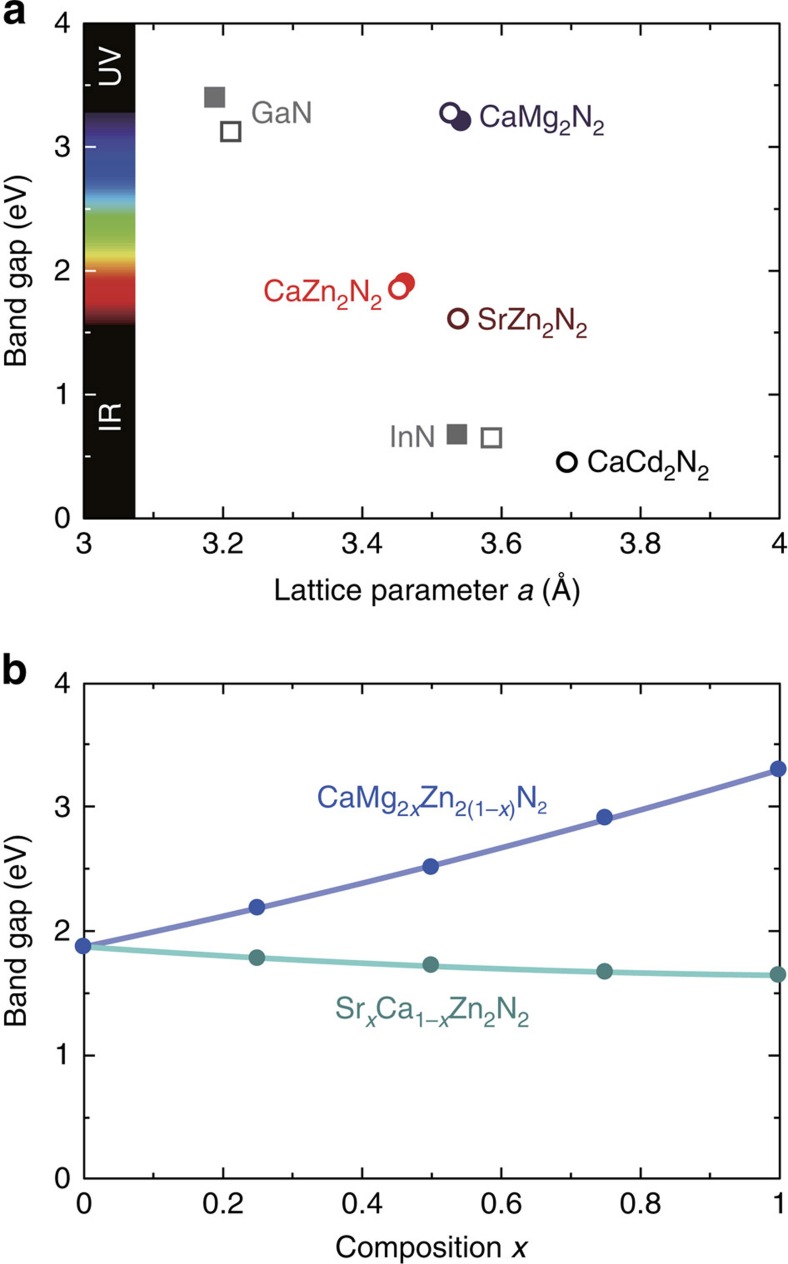
Theoretical bandgaps of nitrides relevant to CaZn_2_N_2_ and its alloys. (**a**) Bandgaps of CaZn_2_N_2_ and relevant isostructural ternary nitrides versus lattice parameter *a*, alongside those of GaN and InN. Theoretical and experimental values are shown with open and filled symbols, respectively; the experimental gap and lattice parameter of CaZn_2_N_2_ are obtained in the present study, whereas the other experimental values are from literature (see Supplementary Tables 4 and 8 for tabulated values and references). (**b**) Theoretical bandgaps of CaMg_2*x*_Zn_2(1−*x*)_N_2_ and Sr_*x*_Ca_1−*x*_Zn_2_N_2_ (0≤*x*≤1) alloys. The curves are quadratic fittings to 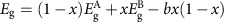, where *E*_g_, 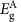 and 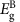 are the bandgaps of the alloy and alloy components A and B, respectively. Band bowing parameters *b* are 0.24 and 0.17 eV for CaMg_2*x*_Zn_2(1−*x*)_N_2_ and Sr_*x*_Ca_1−*x*_Zn_2_N_2_, respectively.

The applications of semiconductors often require tuning of bandgaps, typically via alloying. This is explored for CaZn_2_N_2_ theoretically. CaMg_2_N_2_ (ref. 32), which is a known phase isostructural to CaZn_2_N_2_, is found to be a good alloying agent (Fig. 4a). As expected from a relatively small lattice mismatch (+2.1 and +1.0% against CaZn_2_N_2_ for lattice parameters *a* and *c*, respectively), our cluster expansion and grand canonical Monte Carlo simulations predict alloy formation for the whole composition range of CaMg_2*x*_Zn_2(1−*x*)_N_2_ (0≤*x*≤1) even at room temperature (Supplementary Fig. 11). The bandgap of the CaMg_2*x*_Zn_2(1−*x*)_N_2_ alloy increases almost linearly with the Mg composition from 1.8 eV to the bandgap of CaMg_2_N_2_, 3.3 eV, preserving the direct-type band structure (Fig. 4b). This covers most of the visible light range. The hole and electron effective masses of CaMg_2_N_2_ are 1.8*m*_0_ and 0.2*m*_0_, respectively (Supplementary Table 5). These values, coupled with the similarity in valence and conduction band structures between CaZn_2_N_2_ and CaMg_2_N_2_ (Fig. 2b, right of panel; Supplementary Fig. 4v), imply that the electron effective mass does not change significantly by alloying, whereas the hole effective mass increases up to 1.8*m*_0._ We note that this value is still comparable to the hole effective mass of GaN (2.0*m*_0_).

The bandgap of CaZn_2_N_2_ can also be narrowed towards the near-infrared range. Isostructural SrZn_2_N_2_, which is previously unreported and found to be metastable with respect to decomposition into competing phases (Supplementary Fig. 3f), shows a theoretical bandgap of 1.6 eV (Fig. 4a) and the substitution of Sr for Ca reduces the bandgap of CaZn_2_N_2_ (Fig. 4b). Given the similarity in band structures and carrier effective masses between CaZn_2_N_2_ and SrZn_2_N_2_ (Fig. 2b, right of panel; Supplementary Fig. 4x; Supplementary Table 5), the changes in the effective masses by alloying are expected to be small. The predicted mutual solubility of CaZn_2_N_2_ and SrZn_2_N_2_ is limited at low temperature, compared with the CaZn_2_N_2_–CaMg_2_N_2_ system (Supplementary Fig. 12). Still, quenching from a moderate temperature (∼1,000 K or above) or non-equilibrium growth would allow for bandgap tuning via alloying in a wide composition range. Isostructural BaZn_2_N_2_ was also considered as an alloying agent, but is found to be unstable against lattice vibration (Supplementary Fig. 2y). Another option for bandgap narrowing is the substitution of Cd for Zn. This is expected to reduce the bandgap of CaZn_2_N_2_ substantially, given the theoretical bandgap of 0.4 eV for CaCd_2_N_2_; however, replacing too much Zn with Cd is likely to result in an excessive relaxation that leads to the breakdown of the CaZn_2_N_2_ structure (Supplementary Method 2). In addition, the use of Cd would ideally be avoided in view of the toxicity of many of Cd compounds. The bandgap of CaZn_2_N_2_ is thus most readily tuned via alloying with CaMg_2_N_2_ towards higher energies. We note that the lattice mismatch in the CaZn_2_N_2_–CaMg_2_N_2_ system (∼2%) is much smaller than that of the GaN–InN system (∼10%), as shown in Fig. 4a. This, in turn, allows for the stable alloy formation as discussed above.

### Predictions on the dopability of Ca_2_ZnN_2_ and CaZn_2_N_2_

**Figure 5:**
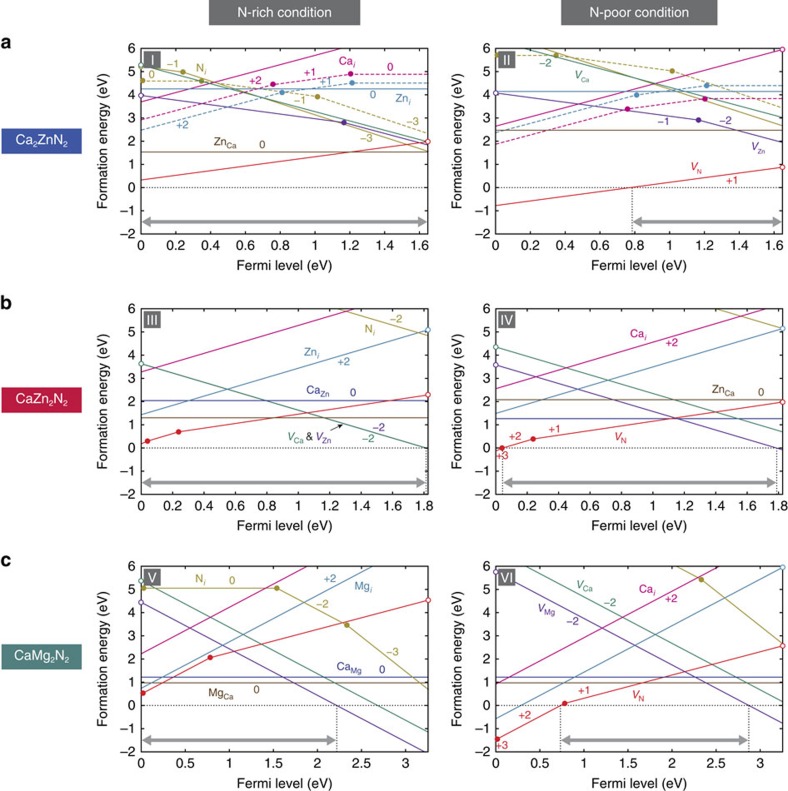
Theoretical defect energetics in Ca_2_ZnN_2_, CaZn_2_N_2_ and CaMg_2_N_2_. The formation energies of native point defects as a function of the Fermi level for (**a**) Ca_2_ZnN_2_, (**b**) CaZn_2_N_2_ and (**c**) CaMg_2_N_2_ under N-rich and N-poor chemical potential conditions, corresponding to I–VI indicated in Fig. 2a and Supplementary Fig. 13. The range of the Fermi level is given by the valence band maximum, which is set to zero, and the conduction band minimum. *V* in defect species denotes vacancies. The subscripts designate defect sites, where *i* means interstitial sites. The results for two interstitial sites in Ca_2_ZnN_2_ are shown with solid and broken lines (Supplementary Fig. 14). The charge states of defects, which correspond to the gradients as defined by equation (1) in the Methods section, are described; only the most energetically favourable charge state at a given Fermi level is shown for each defect. Positive and negative charge states mean donor and acceptor behaviour of defects, respectively. The Fermi level at which the favourable charge state changes corresponds to the positions of donor or acceptor levels, which are designated by filled circles; shallow donor or acceptor levels associated with electronic states inheriting host orbital characteristics are designated by open circles. The controllable ranges of the Fermi level, in which carrier compensation by spontaneous formation of native defects is avoidable, are indicated by arrows.

We now discuss the dopability of Ca_2_ZnN_2_ and CaZn_2_N_2_ into *p* and/or *n* type, including the effect of alloying of CaZn_2_N_2_ with CaMg_2_N_2_, based on the defect chemistry, that is, whether carrier compensation by native defects can be sufficiently suppressed and whether dopants that act as shallow accepters or donors exist. The theoretical defect energetics indicates that dominant donor-type native defects are N vacancies (*V*_N_) in both Ca_2_ZnN_2_ and CaZn_2_N_2_ (Fig. 5a,b). They form shallow donor levels, which are associated with host CBM states perturbed by the N vacancies. The formation energies of the N vacancies are relatively low under N-poor conditions, such as condition II for Ca_2_ZnN_2_ and IV for CaZn_2_N_2_. In particular, the N vacancy shows quite low formation energy in Ca_2_ZnN_2_ even with the Fermi level near the CBM, implying its native *n*-type characteristic when prepared under N-poor conditions. Turning to N-rich conditions, as represented by condition I, allows for the suppression of the N vacancy formation. In connection with this relatively large chemical potential dependence, our Ca_2_ZnN_2_ sample exhibits a semiconducting behaviour with an activation energy of ∼20 meV (Supplementary Fig. 15), whereas a previous study obtained an insulating sample^27^. Among acceptor-type defects, the Ca and Zn vacancies (*V*_Ca_ and *V*_Zn_) are dominant in both nitrides. Interstitial defects (Ca_*i*_, Zn_*i*_ and N_*i*_) generally show high formation energies. Cation antisites are often major defects in ternary compounds, while the formation energies of Ca-on-Zn and Zn-on-Ca antisites (Ca_Zn_ and Zn_Ca_) are not significantly low (>1.2 eV) in both Ca_2_ZnN_2_ and CaZn_2_N_2_. This is presumably because of a relatively large-size mismatch between Ca(II) and Zn(II) ions. More importantly, the isovalence of Ca and Zn results in neutral and, therefore, electrically inactive antisite defects. This leads to a design principle of ternary compound semiconductors: a large-size mismatch and/or the isovalence of two types of cations are desirable to suppress the charge compensation by antisite defects.

The controllable range of the Fermi level is restricted by the spontaneous formation of charged native defects that compensate carriers, that is, their negative formation energies^33^. All defects show positive formation energies in Ca_2_ZnN_2_ under N-rich conditions as represented by condition I, irrespective of the Fermi level position. Similarly in CaZn_2_N_2_, positive formation energies are recognized for almost whole range of the Fermi level under both N-rich (III) and N-poor (IV) conditions. Therefore, electron and hole densities are not significantly limited in terms of native defect compensation, when the Fermi level is controlled towards the VBM or CBM via doping. In other words, both *n*- and *p*-type doping is feasible in both Ca_2_ZnN_2_ and CaZn_2_N_2_. The defect energetics in CaMg_2_N_2_ (Fig. 5c), in contrast, indicates that electron carrier density is limited by the compensation by cation vacancies in CaMg_2*x*_Zn_2(1−*x*)_N_2_ alloys with a high Mg content.

The above assessment of the dopability assumes the availability of dopants that act as shallow acceptors or donors. Our calculations indeed found such dopants having small size mismatches with host ions: Na and K at the Ca site as shallow acceptors and Ga at the Zn site as a shallow donor in Ca_2_ZnN_2_, where Al at the Zn site acts as a deep donor; and Na and K at the Ca site as shallow acceptors, and Al and Ga at the Zn or Mg site as shallow donors in CaZn_2_N_2_ and CaMg_2_N_2_. Another assumption made is a thermodynamic equilibrium. The use of non-equilibrium growth and/or doping conditions can allow for the Fermi level control over the aforementioned limits.

## Discussion

Our high-throughput first-principles screening has identified 10 previously reported and 11 unreported nitrides as promising semiconductors. The latter class of materials includes CaZn_2_N_2_, which has a tunable direct bandgap and small effective masses, is comprised of only abundant elements, and is obtainable via high-pressure synthesis. Other predicted rare-element-free nitrides include newly identified Mg_2_ZnN_2_, ZnTiN_2_ and ZnZrN_2_, as well as Ca_2_ZnN_2_, Sr_2_ZnN_2_, Ba_2_ZnN_2_ and Zn_2_PN_3_, for which synthesis has been reported previously, but the applications as semiconductors have not been suggested to the best of our knowledge. The variety in bandgaps of the identified nitrides, coupled with small effective masses, widens the choice of nitride semiconductors that can be used in electronics, optoelectronics and photovoltaics. The present study demonstrates accelerated materials discovery via computational screening followed by targeted experiments, particularly showing high-pressure synthesis to be effective in the realization of as-yet-unreported nitrides.

## Methods

### Calculations of fundamental properties and stability

The first-principles calculations were conducted using the projector augmented-wave method^34^ and either the Perdew–Burke–Ernzerhof generalized gradient approximation (PBE-GGA) functional^35^ or the Heyd–Scuseria–Ernzerhof (HSE06) hybrid functional^36^,^37^, as implemented in the Vienna Ab initio Simulation Package (VASP)^38^,^39^. The PBE-GGA was used for screening in terms of thermodynamic and lattice dynamic stability. The HSE06 hybrid functional was employed for evaluating the bandgaps, carrier effective masses, electronic band structures, electronic densities of states, absorption spectra and point-defect energetics of the identified compounds. Thermodynamic stability was also investigated using HSE06 and such results for the Ca–Zn–N and Ca–Mg–N ternary systems are shown in Fig. 2a and Supplementary Fig. 13 for consistency with point-defect calculations. The phase diagrams were drawn using the CHESTA code^40^, where competing phases that are reported to be stable in the Materials Project database^41^ are considered. Plane-wave cutoff energies of 550 and 400 eV were used for the geometry optimization of perfect crystals and for the calculations of fundamental and defect properties, respectively. Even *k*-point meshes were used in geometry optimization, which were determined on the basis of the convergence of PBE-GGA total energies: the criterion of the total energy change was set at <0.005 meV per atom per the number of incremental *k* points. For instance, this results in an 8 × 8 × 4 mesh for the wurtzite GaN unit cell. Three times finer meshes were used in the calculations of electronic densities of states and absorption spectra. The bandgaps obtained using the HSE06 hybrid functional are generally close to available experimental values for relevant systems as shown in Supplementary Table 4, alongside comparison with theoretical values from the *GW* approximation^42^ to many-body perturbation theory (see also a discussion in Supplementary Note 2). Effective masses were estimated by quadratic fittings of the bands that constitute the VBM and CBM, using fine sampling of *k* points around the VBM and CBM. The absorption spectra were obtained via the calculations of dielectric functions within the independent particle approximation, where excitonic effects and phonon-assisted absorption were not considered. Details of the screening procedure based on these calculations are described in Supplementary Method 1.

### Crystal structure prediction

The crystal structure search was performed using an evolutionary algorithm as implemented in the USPEX code^12^,^26^, in conjunction with first-principles calculations using the PBE-GGA as described above. The chemical formula for each system was fixed, but allowed to vary in multiples of between 1 and 4. The calculations were considered converged when no lower energy structure could be found after 20 generations; with each generation containing 20 individual structures.

### Alloy property calculations

The phase diagrams of the CaZn_2_N_2_–CaMg_2_N_2_ and CaZn_2_N_2_–SrZn_2_N_2_ pseudobinary systems were constructed using the PBE-GGA and the cluster expansion technique^43^,^44^ in conjunction with grand canonical Monte Carlo simulations, using the CLUPAN code^45^. Quasi-random structures^46^ were employed for predicting band bowing using the HSE06 hybrid functional. More details of the alloy property calculations are given in Supplementary Method 2 and Supplementary Figs 16–21.

### Modelling of point defects

Native point defects and dopants in Ca_2_ZnN_2_, CaZn_2_N_2_ and CaMg_2_N_2_ were modelled using 90-atom supercells (Supplementary Fig. 14). Atomic positions were relaxed with the cell parameters fixed at the perfect crystal values. Spin polarization was considered for odd-numbered electron systems. Defect formation energies were evaluated as^47^


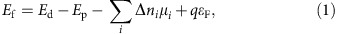


where *E*_d_ is the total energy of a supercell containing a defect in charge state *q*, *E*_p_ is that of the perfect crystal supercell and Δ*n*_*i*_ is the difference in the number of constituent atom *i* between these supercells. *μ*_*i*_ and *ɛ*_F_ are the chemical potential of atom *i* and the Fermi level, respectively. *E*_d_ and *E*_p_ are evaluated using the HSE06 hybrid functional. The finite-size effects of supercells associated with electrostatic interactions between charged defects, their periodic images and the charge compensating background were corrected using the scheme reported in refs 48, 49. Our implementation accounts for the anisotropic screening of defect charges using dielectric tensors. In addition, atomic-site local potential is used as a potential marker, which has been shown to be effective for relaxed atomic configurations^49^.

### Synthesis of Ca_2_ZnN_2_ and CaZn_2_N_2_

Polycrystalline Ca_2_ZnN_2_ and CaZn_2_N_2_ disks and powders were prepared by solid-state reactions. Two kinds of common precursor nitrides, Ca_3_N_2_ and Zn_3_N_2_, were employed in both cases. Ca_3_N_2_ was synthesized by heating dendritic pieces of Ca metal (purity: 99.99%, Sigma-Aldrich Co. LLC.) in an electric furnace, directly connected with a glove box filled with dry inert Ar gas (dew point less than −90 °C, oxygen concentration<1 p.p.m.), at 900 °C for 10 h under N_2_ gas (purity: >99.9998 vol% (G2)) flow atmosphere. For Zn_3_N_2_, a commercially available reagent powder (purity: 99%, Alfa Aesar Co.) was employed.

To obtain high-purity Ca_2_ZnN_2_, the precursor powders were mixed in the glove box at the molar ratio of Ca_3_N_2_:Zn_3_N_2_=2.0:1.0 and then the mixture was uniaxially pressed into a disk (6 mm in diameter and ca. 10 mm in height), which was covered by a BN crucible and sealed in an Ar-filled stainless tube as shown in Fig. 3a. The BN crucible prevents chemical reaction between the pressed disk and the stainless tube. The stainless tube was heated at 680 °C (temperature as used in ref. 27) for 40 h. For the synthesis of CaZn_2_N_2_, we employed a belt-type high-pressure apparatus developed by Fukunaga *et al*.^50^. The precursor powders were mixed in the glove box at the molar ratio of Ca_3_N_2_:Zn_3_N_2_=1.0:2.0. The mixture was set up in a high-pressure cell composed of NaCl (10 wt% ZrO_2_) and BN with a carbon heater as shown in Fig. 3b. Pressure was increased to 5.0 GPa for ca. 20 min at room temperature. Temperature was then elevated to 1,200 °C in 30 min and retained for 1 h. After the high-pressure/temperature treatment, the high-pressure cell was water-cooled to room temperature and then returned to ambient pressure. We slightly polished the sample pellet by a file to obtain an unreacted and fresh bulk region of CaZn_2_N_2_ because the top surface of the pellet chemically reacted with a BN surface. The final size of the pellet was 5.5 mm in diameter and ca. 3 mm in height.

CaZn_2_N_2_ is air-stable: any decomposed and impurity phases are not observed by X-ray diffraction, when the sample is exposed at room temperature in air for 1 week. It is stable up to at least 300 °C in air and 500 °C in Ar atmosphere; oxidization occurs at 400 °C in air, and CaZn_2_N_2_ decomposes into Ca_2_ZnN_2_ at 600 °C in Ar atmosphere. On the other hand, Ca_2_ZnN_2_ rapidly decomposes into some oxides and hydroxides, such as ZnO and Ca(OH)_2_ in air.

### Characterization

Crystalline phases were determined by X-ray diffraction using a Cu Kα_1_+Kα_2_ source radiated from a rotary anode (45 kV × 360 mA, to detect a small amount of impurity phases) and a *θ*-coupled 2*θ* scan with Bragg–Brentano geometry. We used an Ar-filled O-ring-sealed sample holder in the X-ray diffraction measurements for exact assessment of formed crystalline phases. Lattice parameters were determined using the Pawley method. Structure refinement was performed using the Rietveld method. These analyses were made using TOPAS Version 4.2 (Karlsruhe, Germany: Bruker AXS). X-ray diffraction profile simulations were conducted using the RIETAN-FP code^51^. Chemical composition, that is, atomic ratio of Ca to Zn, was evaluated with an electron probe micro-analyzer employing a wavelength-dispersive spectroscopy mode.

Diffuse reflectance (*R*) spectra of the polycrystalline samples were measured in the visible–near-infrared wavelength region using a conventional spectrophotometer. To estimate the optical bandgaps, the observed diffuse reflectance spectra were converted by the Kubelka–Munk equation (1–*R*)^2^/(2*R*)=*α*/*S*, where *α* and *S* denote an absorption coefficient and a scattering factor, respectively. Photoluminescence spectra were measured using excitation by third harmonic generation of Nd:YAG pulsed laser (wavelength: 355 nm) with an energy density of ∼7 mJ cm^−2^.

### Data availability

The authors declare that the data supporting the findings of this study are available within the article and its Supplementary Information file.

## Additional information

**How to cite this article**: Hinuma, Y. *et al*. Discovery of earth-abundant nitride semiconductors by computational screening and high-pressure synthesis. *Nat. Commun.* 7:11962 doi: 10.1038/ncomms11962 (2016).

## Supplementary Material

Supplementary Information Supplementary Figures 1-21, Supplementary Tables 1-8, Supplementary Notes 1 and 2, Supplementary Methods 1 and 2 and Supplementary References
