# Supplementary material for: Discovery of earth-abundant nitride semiconductors by computational screening and high-pressure synthesis
Source: Nat Commun. 2016 Jun 21;7:11962. doi: 10.1038/ncomms11962 (PMC4919542; doi:10.1038/ncomms11962)
Supplement: Supplementary Information — Supplementary Figures 1-21, Supplementary Tables 1-8, Supplementary Notes 1 and 2, Supplementary Methods 1 and 2 and Supplementary References [file ncomms11962-s1.pdf]

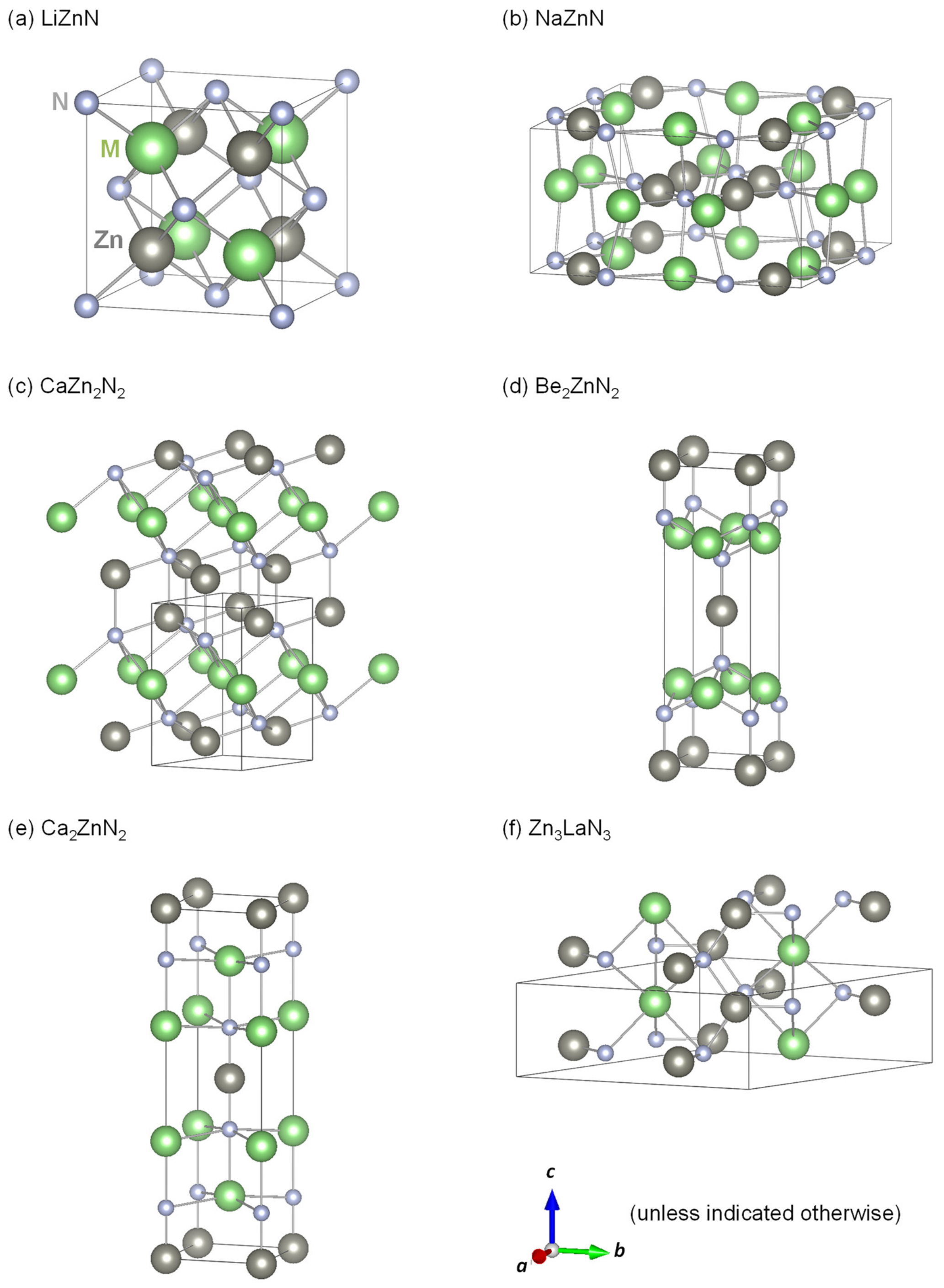

Supplementary Figure 1 (pages 1 and 2). Crystal structures of the identified nitrides. Structures indicated with an asterisk are predicted using an evolutionary algorithm. When more than one nitride takes the same crystal structure, only one representative is shown here (See Supplementary Table 3). The VESTA code[1] was used for illustration.

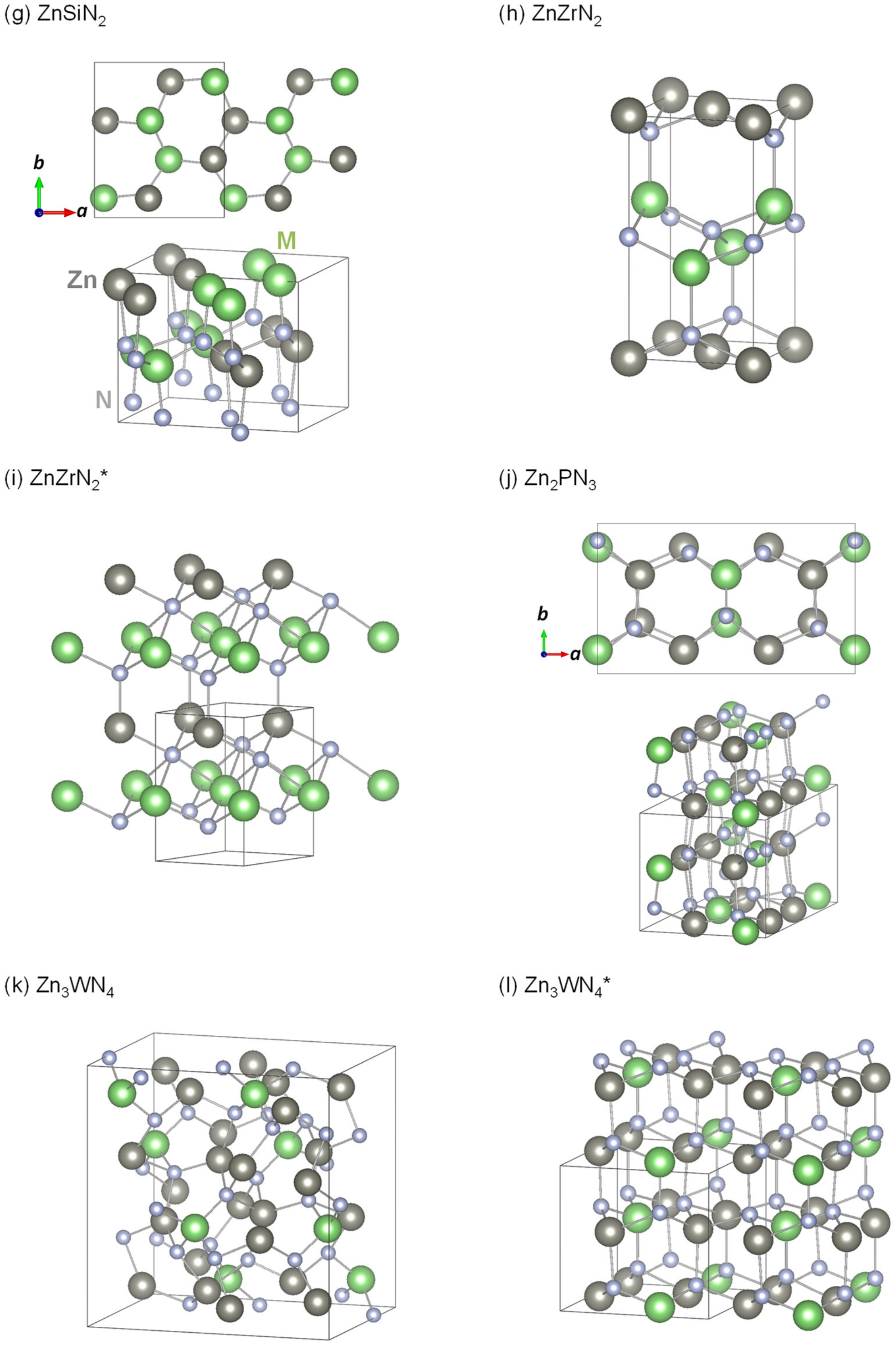

Supplementary Figure 1. (*Continued.*)

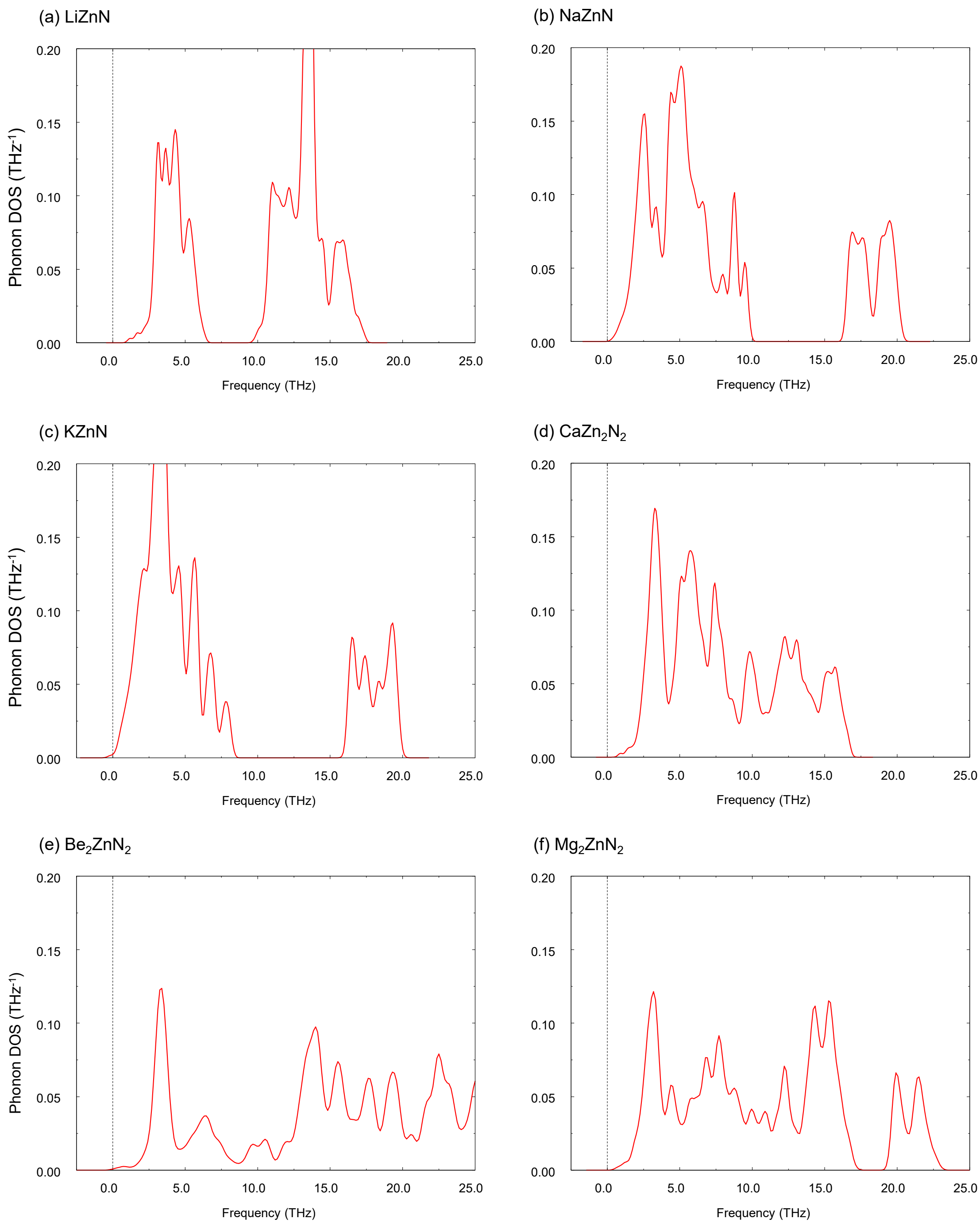

Supplementary Figure 2 (pages 3-7). Phonon DOS of the identified nitrides and derivative compounds of $CaZn_2N_2$ obtained using PBE. Note that a derivative compound, $BaZn_2N_2$, shows an imaginary mode (frequency less than 0 THz) in (y), indicating its dynamic instability; the others are dynamically stable.

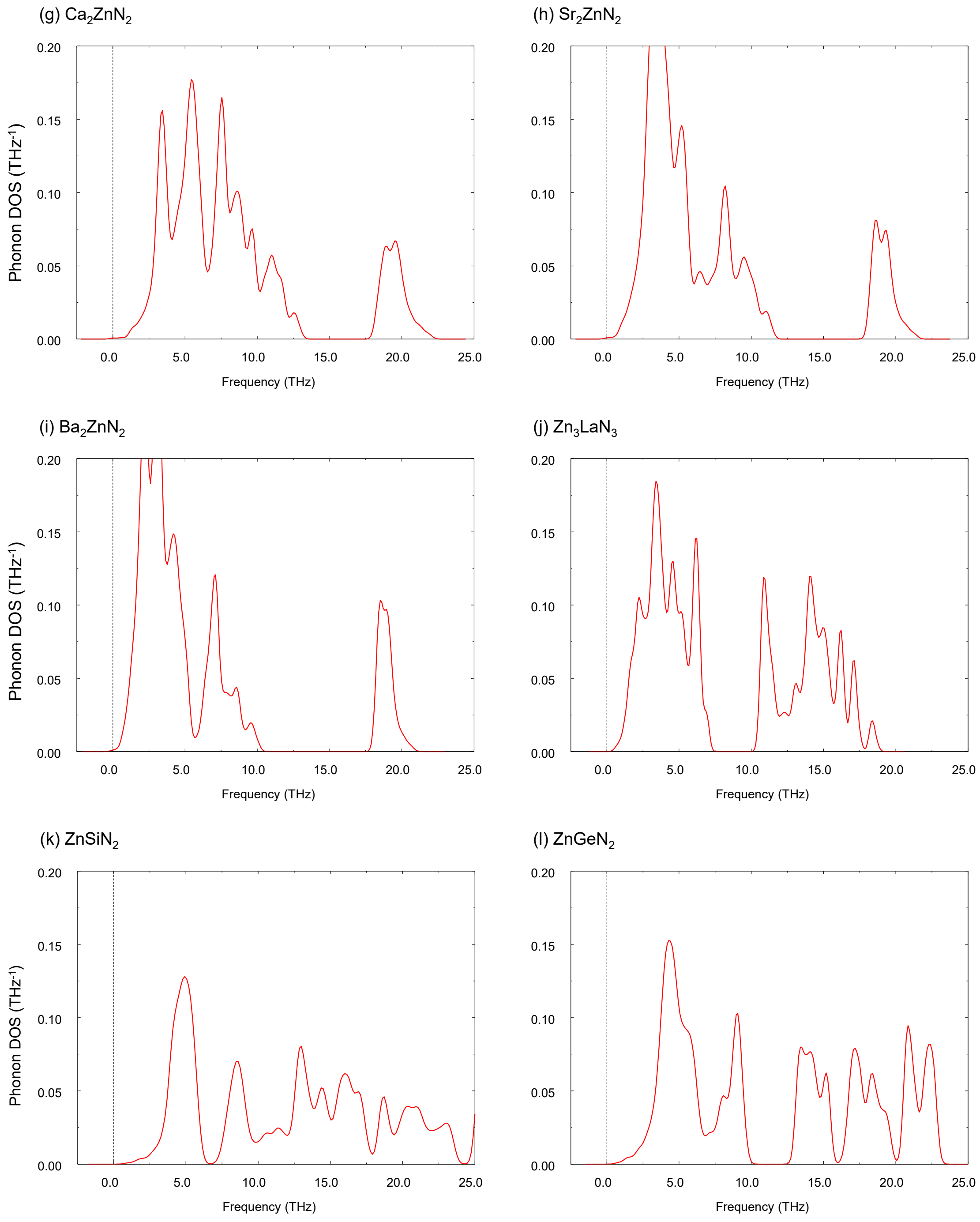

Supplementary Figure 2. (*Continued.*)

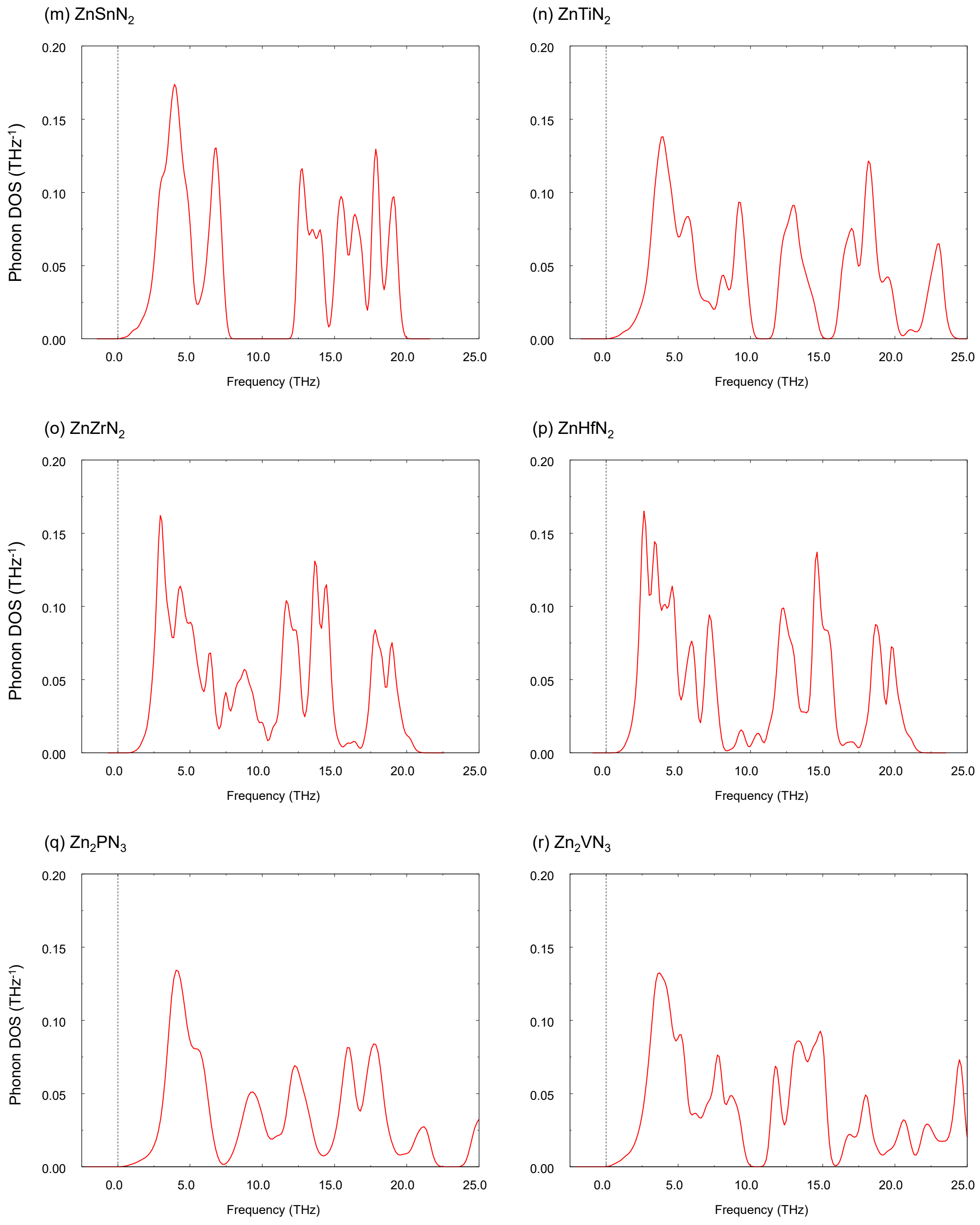

Supplementary Figure 2. (*Continued.*)

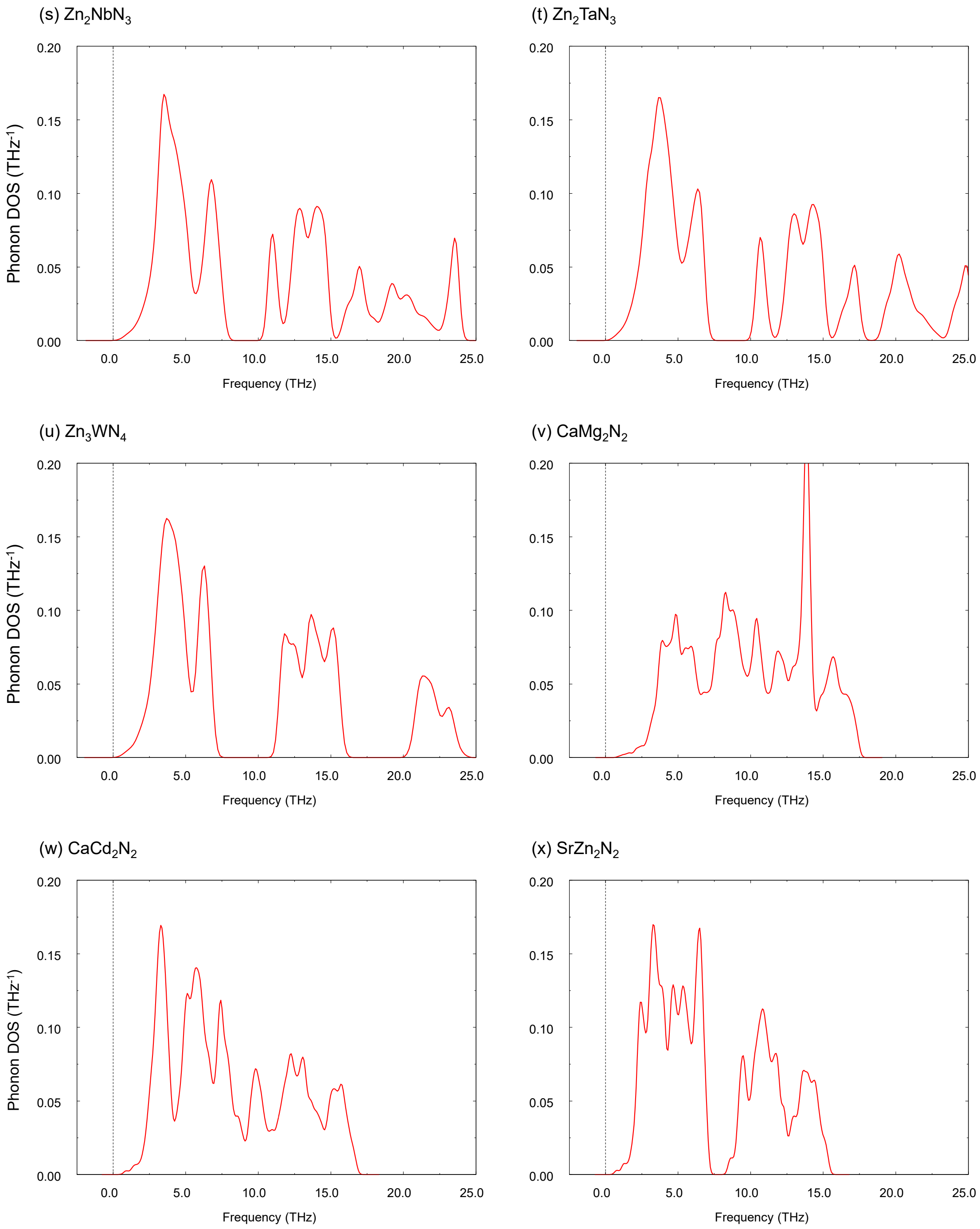

Supplementary Figure 2. (*Continued.*)

(y) $BaZn_2N_2$

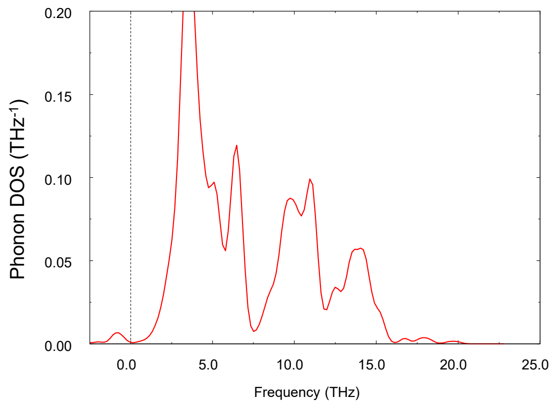

Supplementary Figure 2. (*Continued.*)

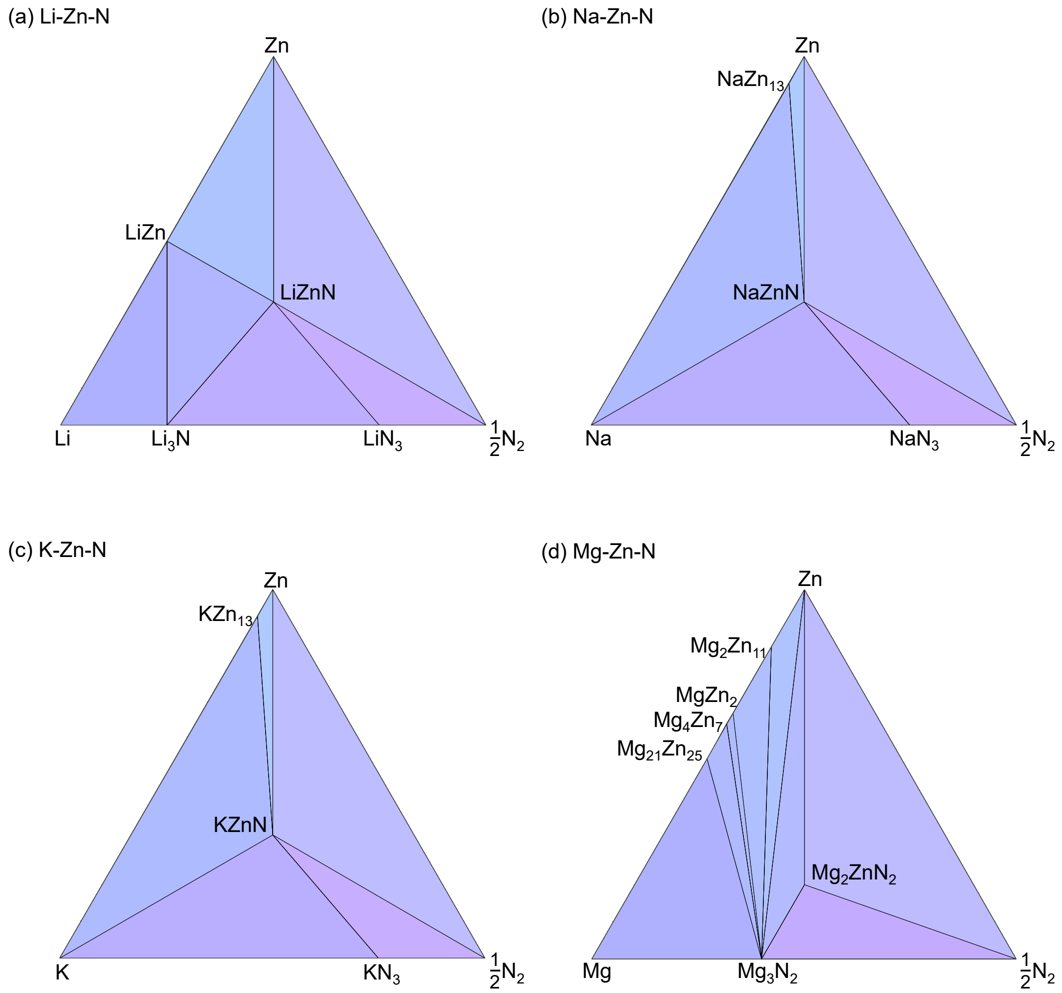

Supplementary Figure 3 (pages 8-11). Ternary phase diagrams of relevant systems obtained using PBE. The Be-Zn-N, Zn-Si-N, Zn-Ge-N, and Zn-Sn-N diagrams are not presented as no competing phases other than Be_3N_2, Si_3N_4, Ge_3N_4, Sn_3N_4, and the simple substances are reported in these ternary systems. The Ca_5Zn_3 phase is stable with PBE but unstable with HSE06; therefore, this phase does not appear in Fig. 2(a) in the main article. Among the 21 identified nitrides, Be_2ZnN_2, Zn_3LaN_3, $ZnSnN_2$, and $ZnTiN_2$ are slightly metastable against phase separation with PBE (with formation energies that are less than 50 meV atom^{-1}). Among these four nitrides, only Be_2ZnN_2 is metastable with HSE06. The diagrams were drawn using the CHESTA code[2].

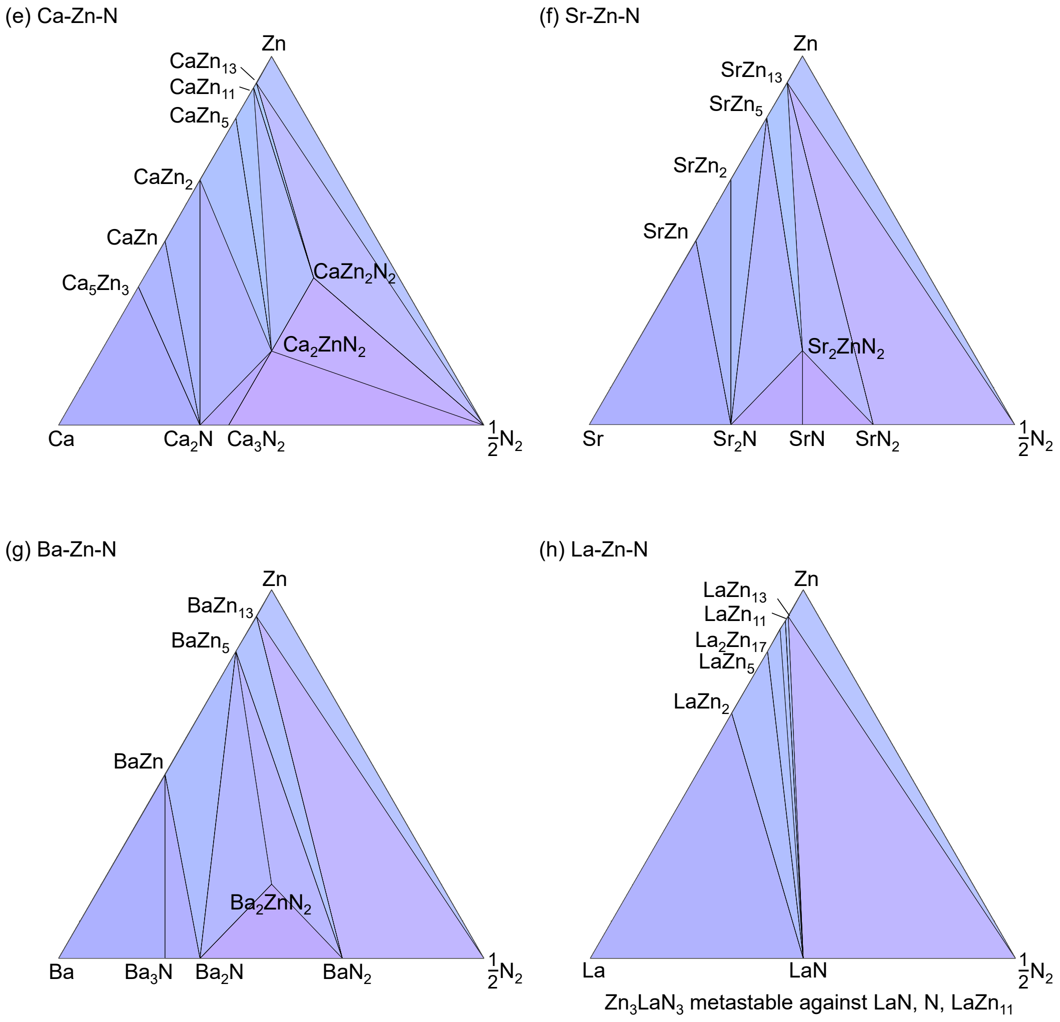

Supplementary Figure 3. (*Continued.*)

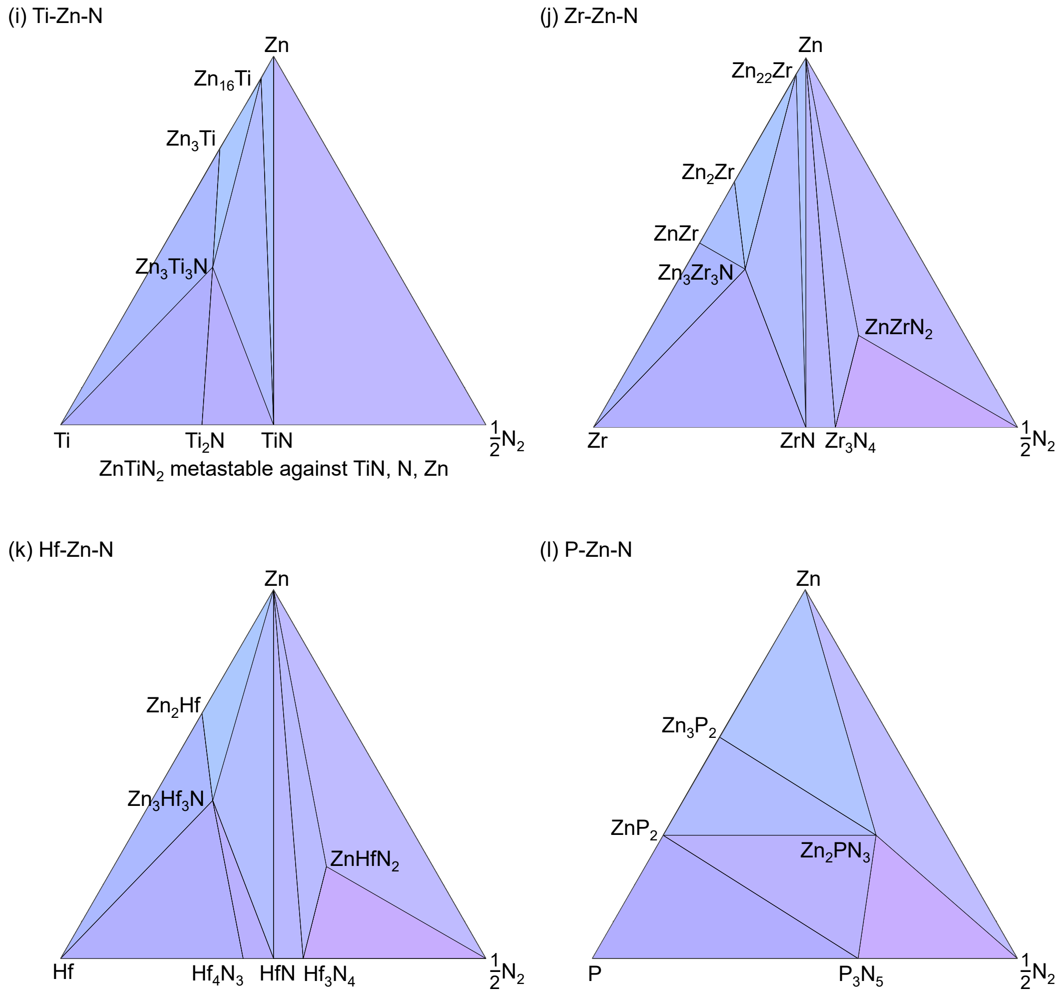

Supplementary Figure 3. (*Continued.*)

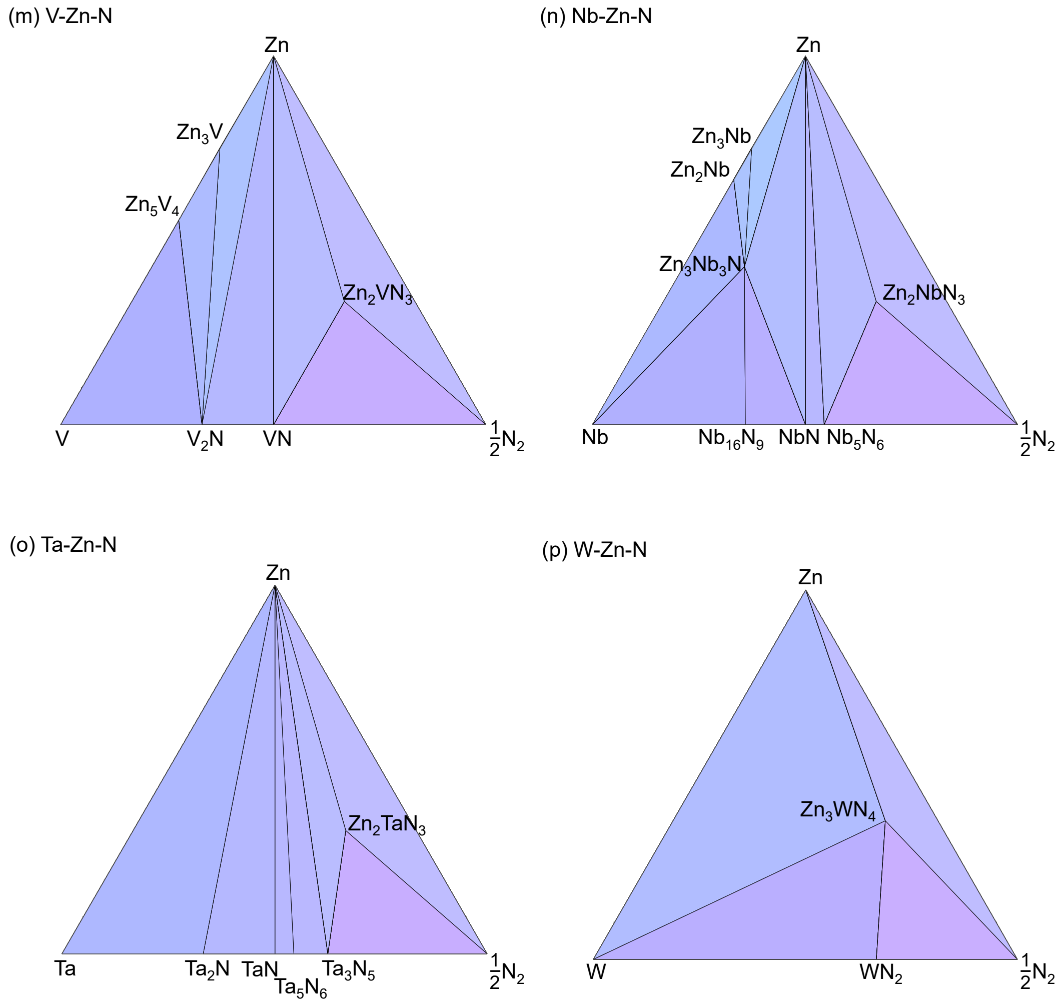

Supplementary Figure 3. (*Continued.*)

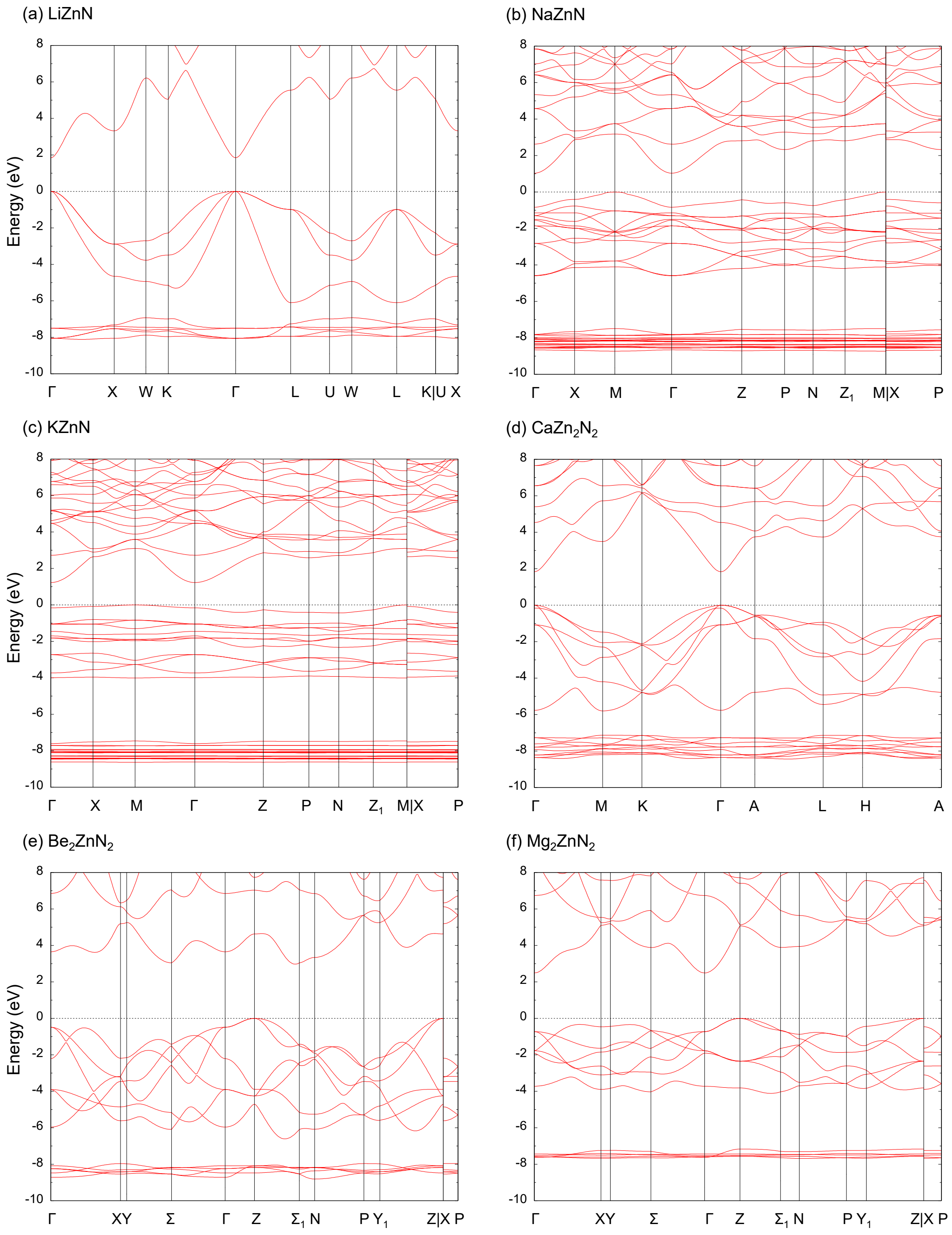

Supplementary Figure 4 (pages 12-16). Band structure diagrams for the identified nitrides and their relevant nitrides obtained using HSE06. Points in the Brillouin zone are labeled based on the standard cell defined by Setyawan and Curtarolo[3].

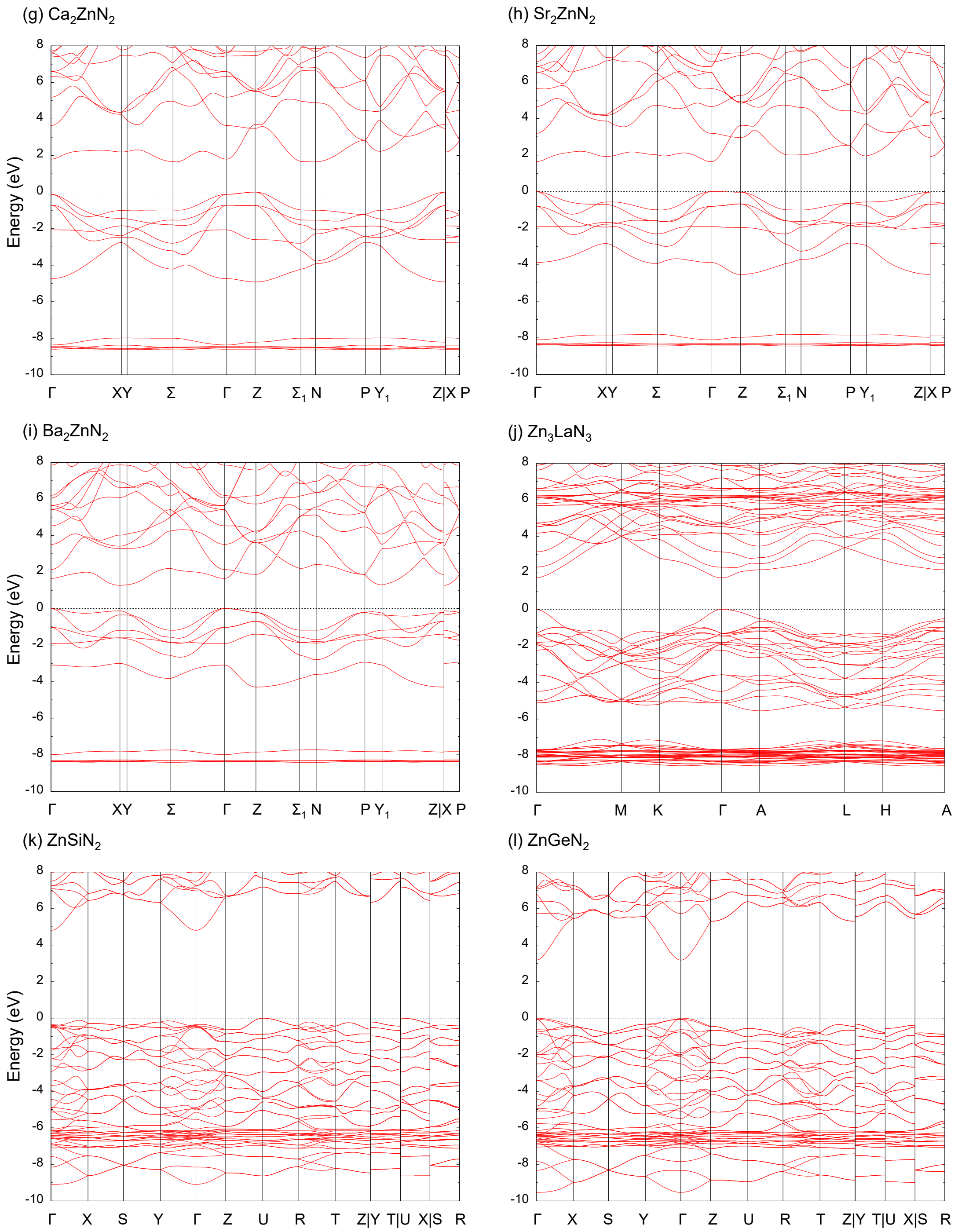

Supplementary Figure 4. (*Continued.*)

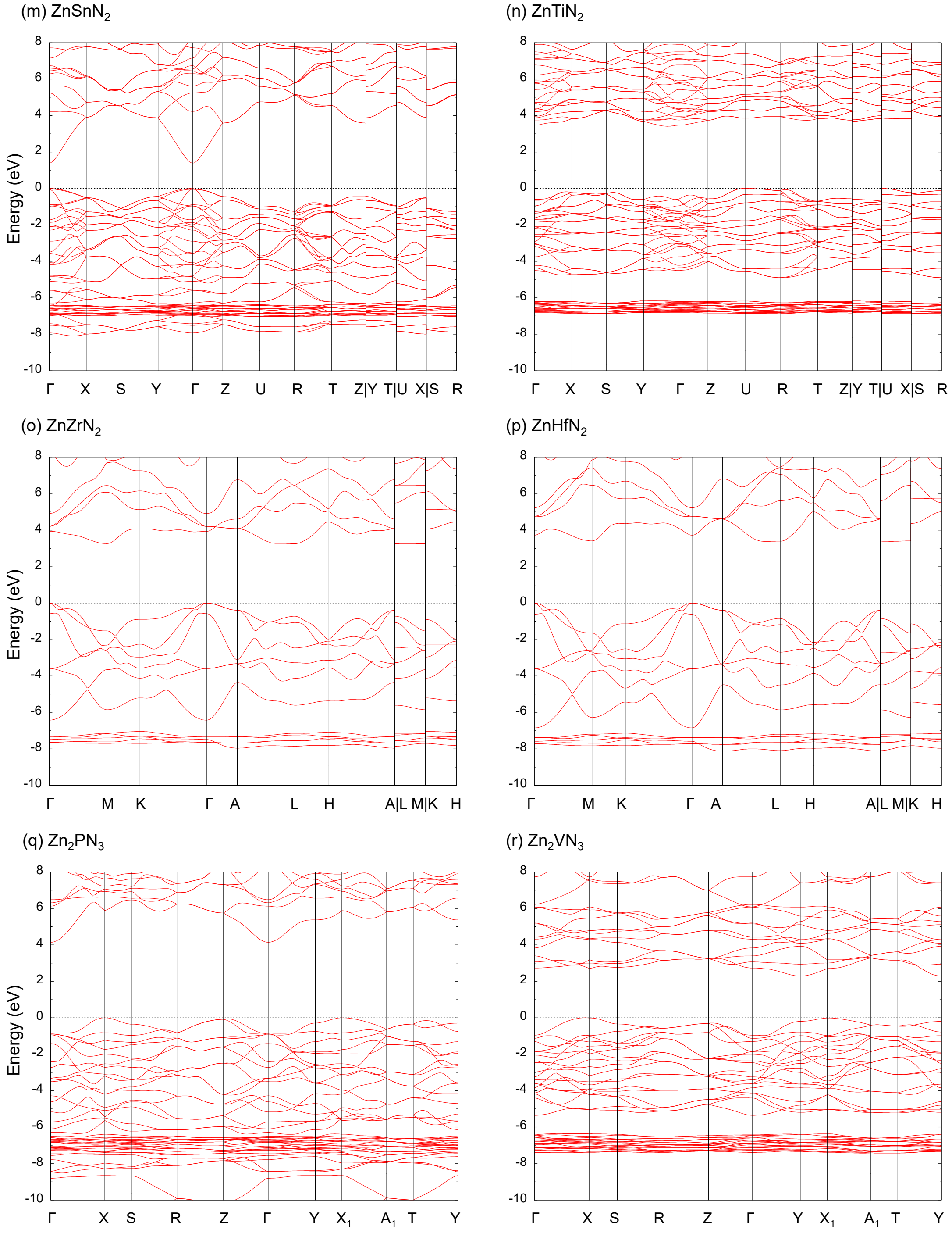

Supplementary Figure 4. (*Continued.*)

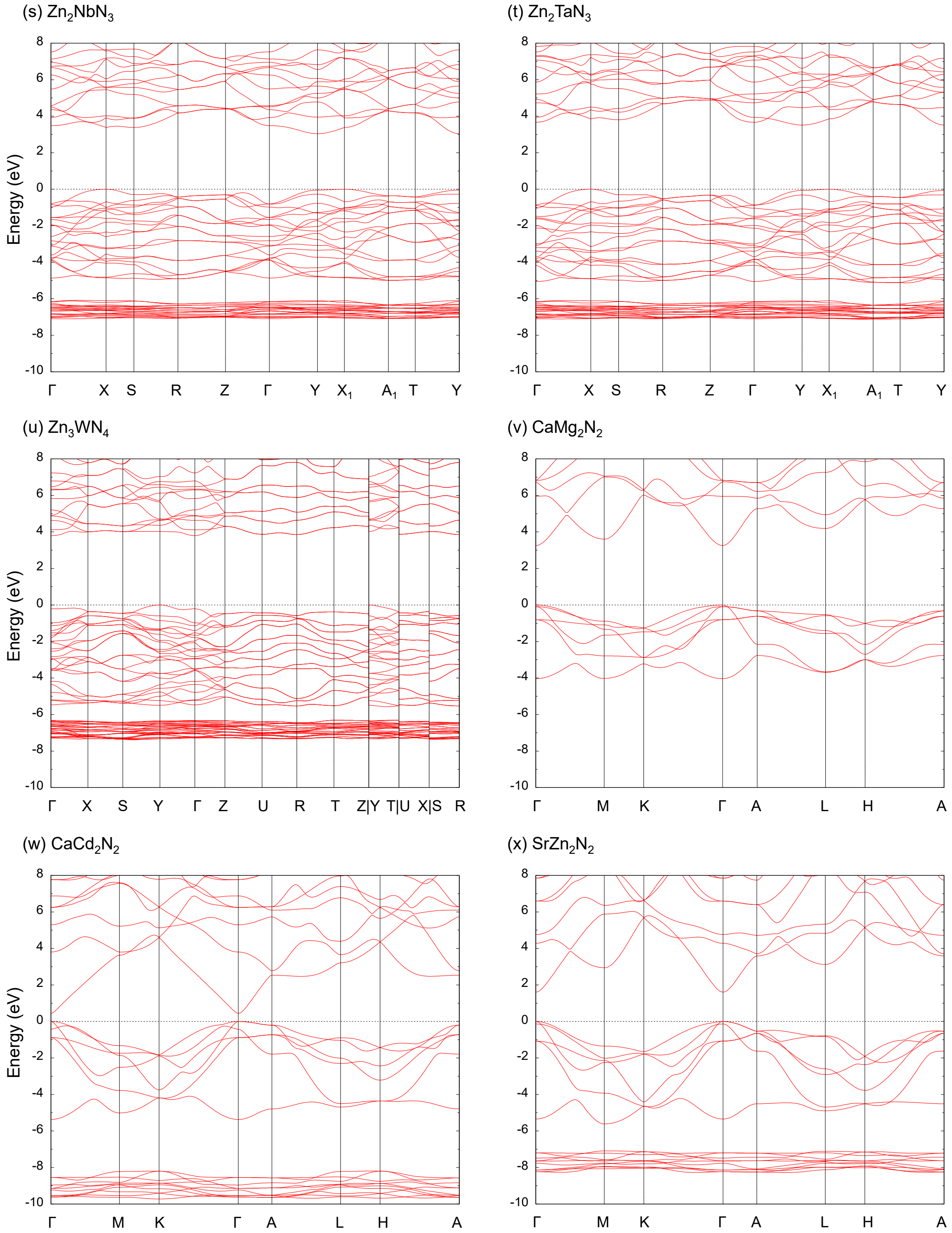

Supplementary Figure 4. (*Continued.*)

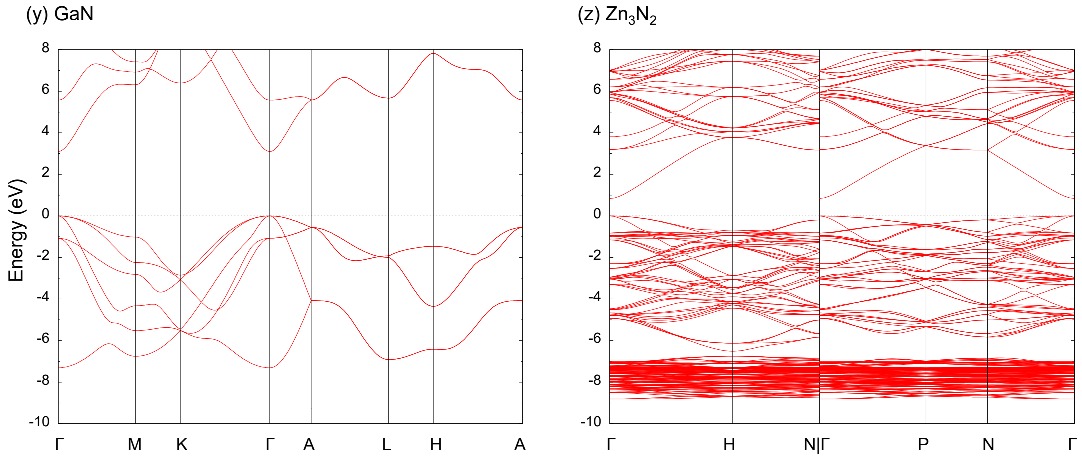

Supplementary Figure 4. (***Continued.***)

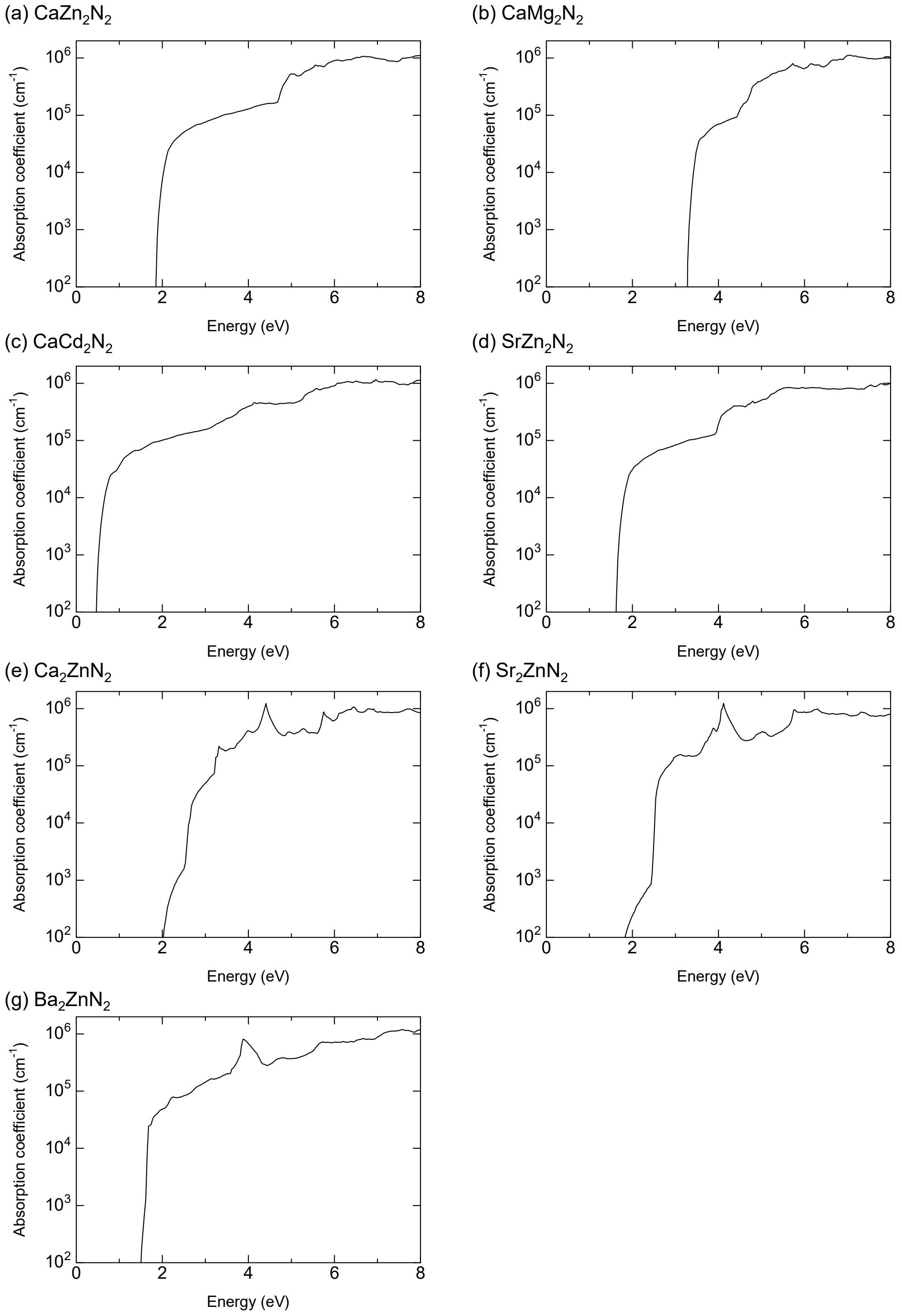

Supplementary Figure 5. Absorption spectra for selected nitrides obtained using HSE06. $CaMg_2N_2$, $CaCd_2N_2$, and $SrZn_2N_2$, which are derivative compounds of $CaZn_2N_2$, have a direct-type band structure and show steep absorption thresholds at the energies corresponding to their band gaps. Ba_2ZnN_2 also exhibits a steep threshold although it has an indirect-type band structure.

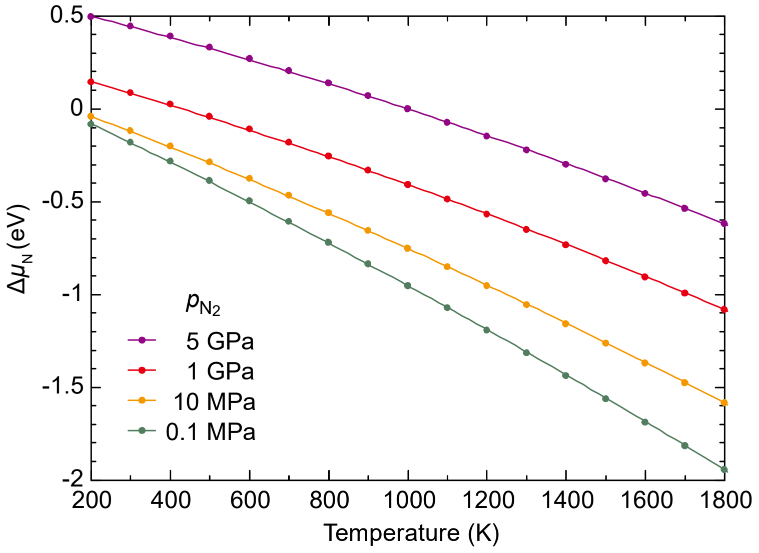

Supplementary Figure 6. Dependence of the N chemical potential on temperature and N_2 partial pressure. The values are obtained using the ideal gas model[4] in conjunction with a fugacity coefficient estimated at high temperature and high pressure[5].

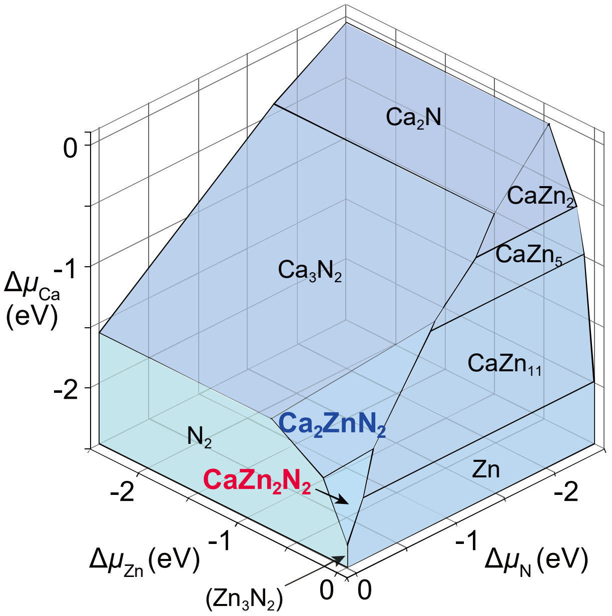

Supplementary Figure 7. Theoretical Ca-Zn-N ternary chemical potential diagram at 0 K and 5 GPa. Enthalpies under these temperature and pressure conditions, obtained using HSE06, were used to determine the chemical potentials of respective solid phases. The diagram was drawn using the CHESTA code[2]. The chemical potentials $\Delta\mu_i$ (i = Ca, Zn, and N) are relative to those at the standard states, which are taken to be the Ca and Zn metals at 0 K and 5 GPa and the N_2 molecule at 0 K and 0 GPa. The Zn_3N_2 phase is metastable and indicated in parenthesis. Compared with the result at 0 GPa [Fig. 2(a) in the main article], it is found that the stable region of the $CaZn_2N_2$ phase is not significantly affected by a pressure of 5 GPa. This is because pressure effects mostly cancel out between the relevant solid phases.

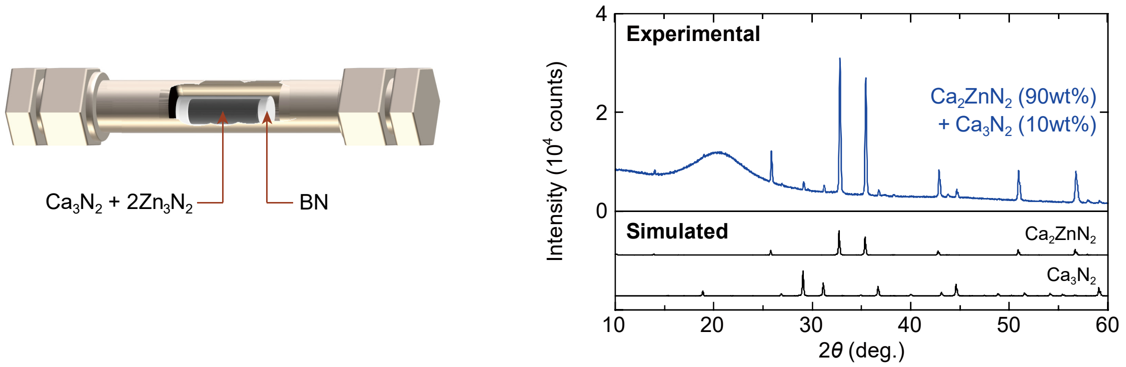

Supplementary Figure 8. Example of X-ray diffraction profiles for samples with a starting composition of Ca:Zn:N = 1:2:2 that were prepared using the method for the synthesis of Ca_2ZnN_2. Schematic of an experimental setup (left of panel) and X-ray diffraction profile (right of panel) for a sample, which was encapsulated in a sealed steel tube and annealed at 680 °C for 62 h. No $CaZn_2N_2$ phase has been obtained with this approach although various annealing conditions were considered.

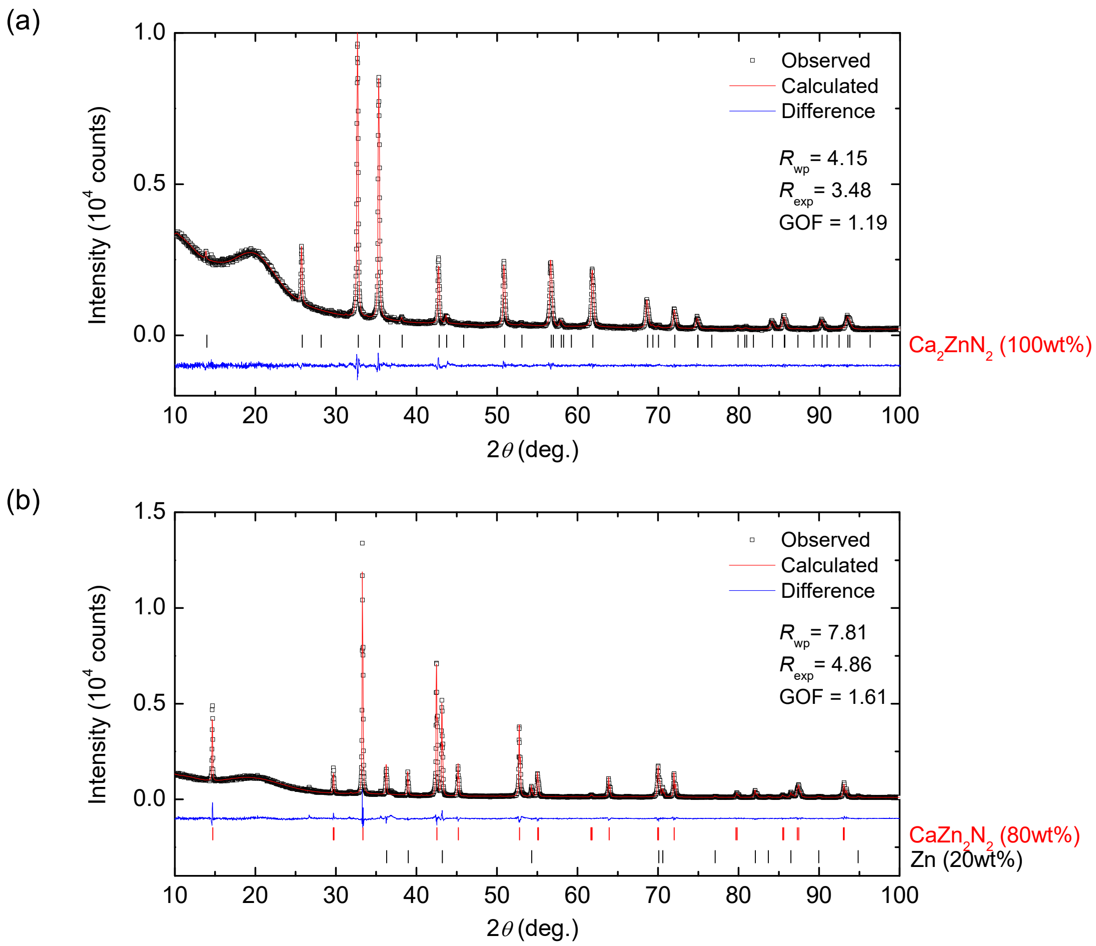

Supplementary Figure 9. Rietveld analysis of Ca-Zn-N samples. (a) Polycrystalline sample with a starting composition of Ca:Zn:N = 2:1:2 aiming at the formation of the Ca_2ZnN_2 phase, which was encapsulated in a sealed steel tube and annealed at 680 °C for 40 h. The obtained reliability factors such as R_{wp}, R_{exp}, and GOF ($=R_{wp}/R_{exp}$) are sufficiently small (see Supplementary Table 6), indicating that the structure model is the same as that reported previously[6]. (b) Polycrystalline sample treated at 1200 °C and 5.0 GPa for 1 h with a starting composition of 1:2:2 corresponding to $CaZn_2N_2$. The Rietveld quantitative analysis indicates that this sample consists of the $CaZn_2N_2$ phase of 80wt% and the Zn phase of 20wt%.

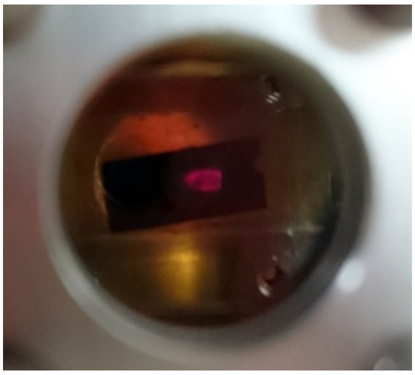

Supplementary Figure 10. Photograph of red photoluminescence from the $CaZn_2N_2$ sample at 300 K.

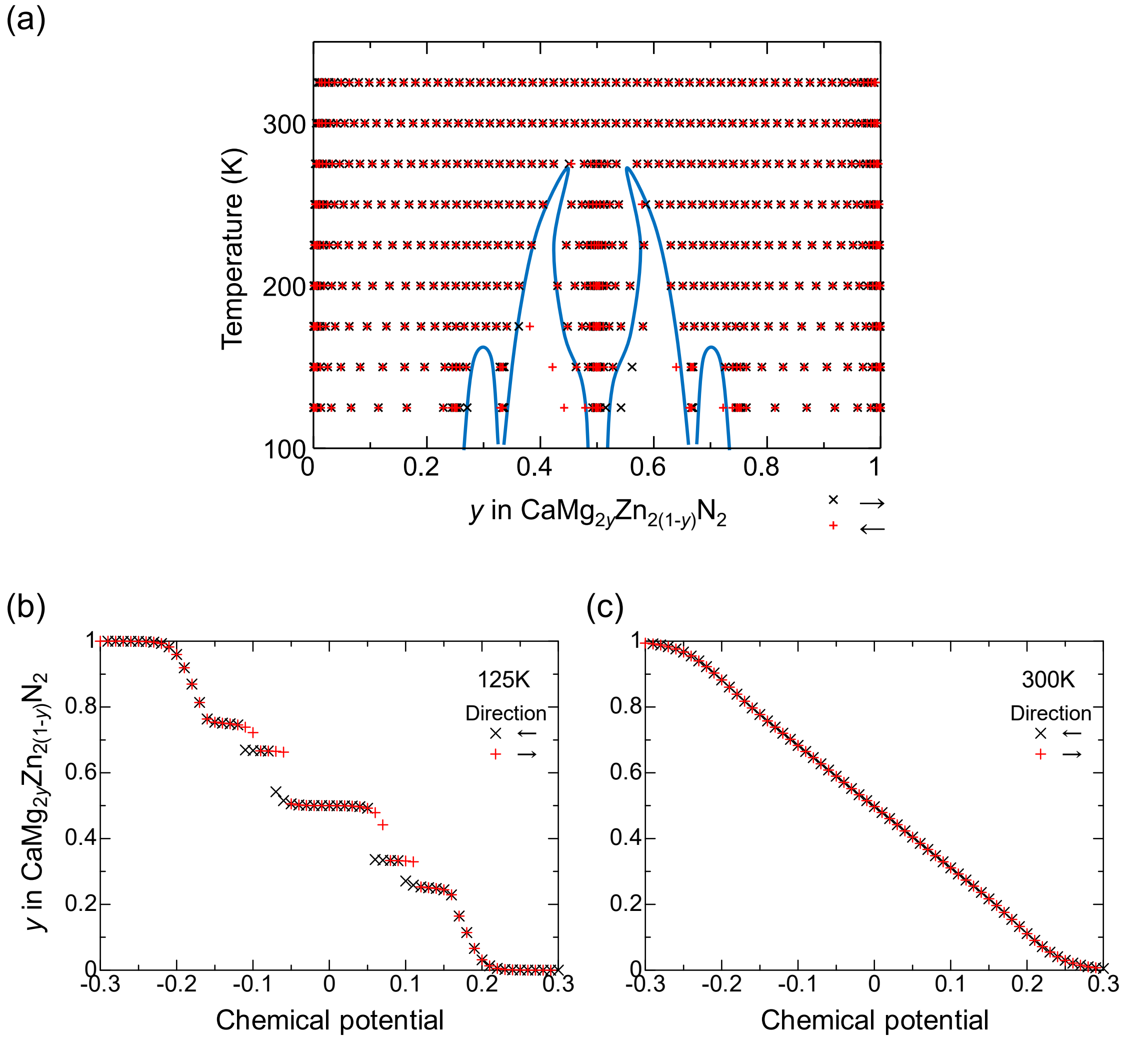

Supplementary Figure 11. Phase diagram of the $CaZn_2N_2$-$CaMg_2N_2$ pseudo-binary system. (a) Composition-temperature diagram. Points are composition-temperature combinations obtained from Monte Carlo simulations at fixed temperature. The blue solid curves indicate estimated phase boundaries. Composition versus chemical potential in simulations at (b) 125 K and (c) 300 K. Black and red crosses correspond to simulations where the chemical potential is gradually decreased and increased, respectively.

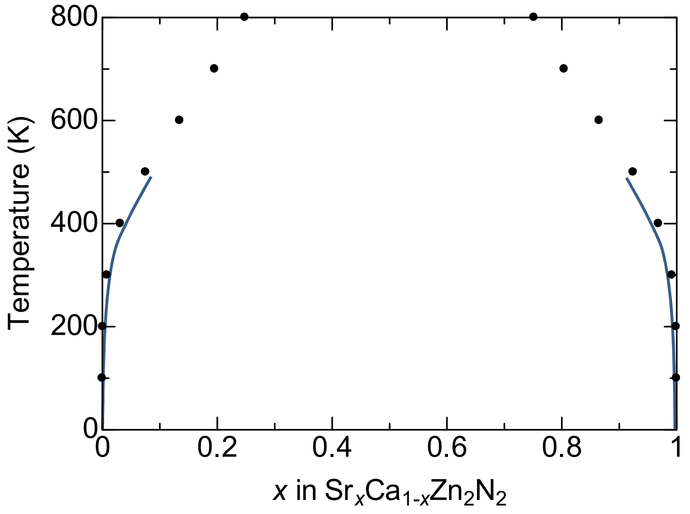

Supplementary Figure 12. Phase diagram of the $CaZn_2N_2$-$SrZn_2N_2$ pseudo-binary system. Points are composition-temperature combinations obtained from Monte Carlo simulations. The solid curves indicate estimated phase boundaries in a low temperature region.

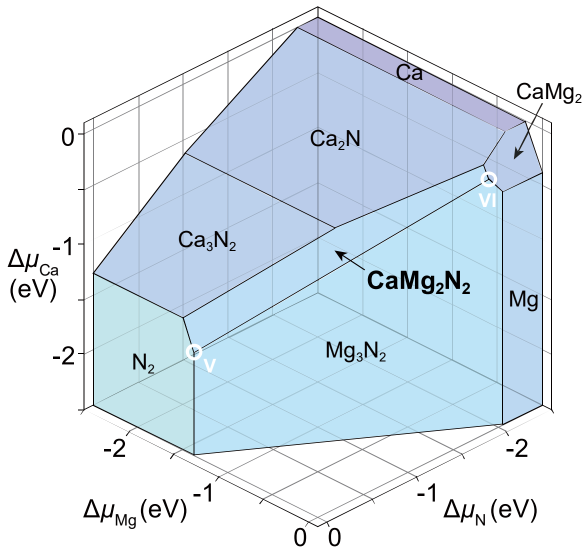

Supplementary Figure 13. Theoretical Ca-Mg-N ternary chemical potential diagram at 0 K and 0 GPa, obtained using HSE06. The chemical potentials $\Delta\mu_i$ (i = Ca, Mg, and N) are relative to those at the standard states, which are taken to be the Ca and Mg metals and the N_2 molecule. Chemical potential conditions V and VI, which are N-rich and N-poor conditions, respectively, are considered in Fig. 5(c) in the main article. The diagram was drawn using the CHESTA code[2].

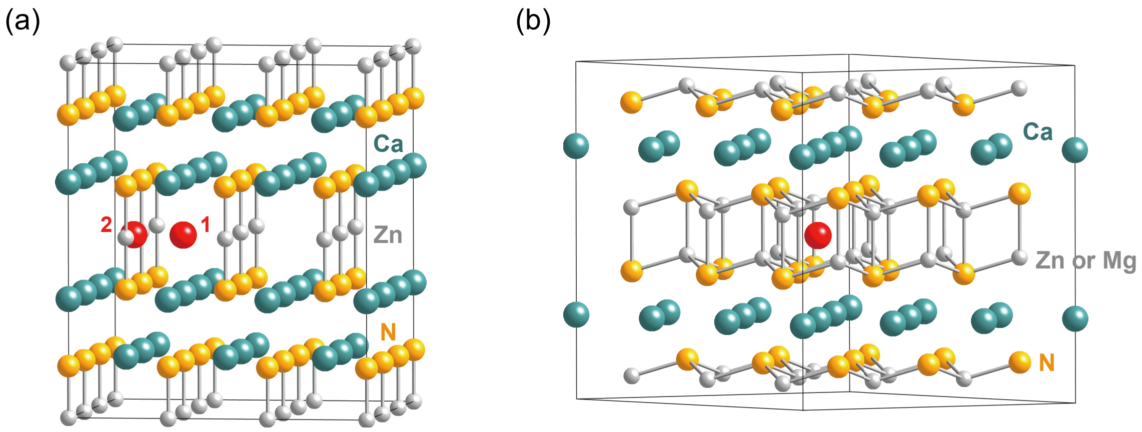

Supplementary Figure 14. Supercells used in the point defect calculations for (a) Ca_2ZnN_2 and (b) $CaZn_2N_2$ and $CaMg_2N_2$. Only Zn-N bonds are shown. Interstitial sites are indicated with red spheres. The results for interstitial sites 1 and 2 in Ca_2ZnN_2 are shown with solid and dashed lines, respectively, in Fig. 5(a) in the main article.

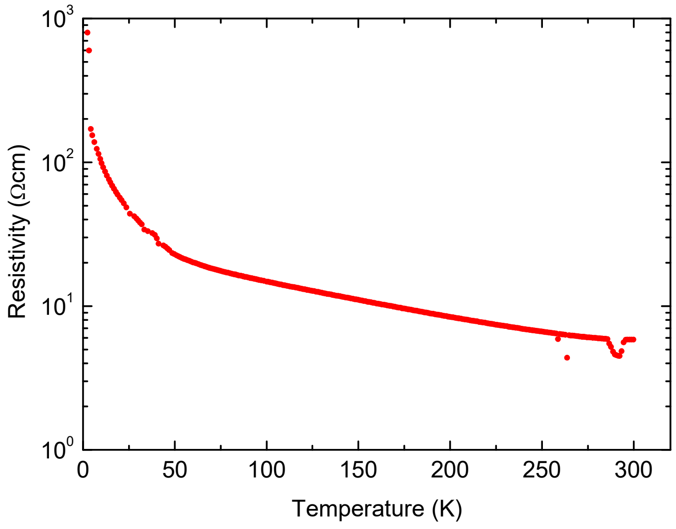

Supplementary Figure 15. Temperature dependence of the electrical resistivity of the Ca_2ZnN_2 sample. Transverse electrical resistivity ρ_{xx} [= 1 / (electrical conductivity σ)] of the Ca_2ZnN_2 sample is shown in the temperature range of 2–300 K. Semiconducting behavior is observed; the carrier activation energy estimated in a temperature range around room temperature is ~20 meV. Ohmic Au electrodes were used in the measurement, which were formed by *in-situ* transferred pulsed laser deposition without exposure to air. Because we could not remove Zn metal impurities in $CaZn_2N_2$ samples, only Ca_2ZnN_2 was investigated.

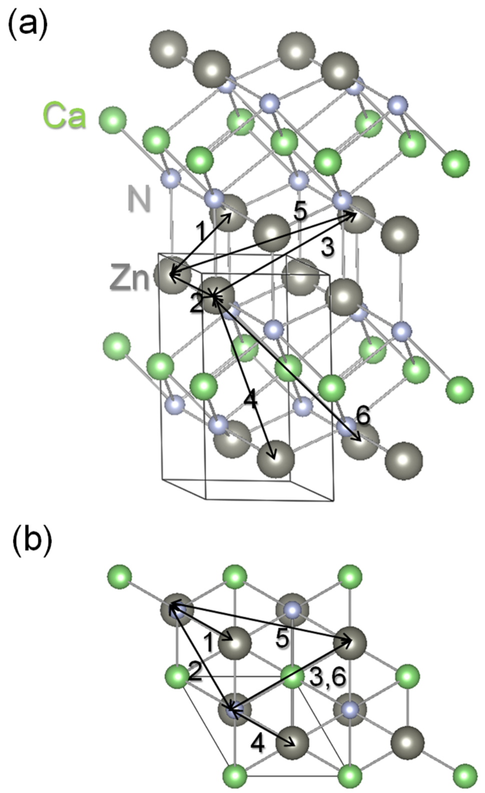

Supplementary Figure 16. Unit cell of $CaZn_2N_2$. (a) View from a direction nearly perpendicular to the *c*-axis. (b) View from the direction parallel to the *c*-axis. Pair clusters considered in the cluster expansion of $CaMg_{2y}Zn_{2(1-y)}N_2$ are indicated (number *n* corresponds to the *n*-th nearest neighbor). The VESTA code[1] was used for illustration.

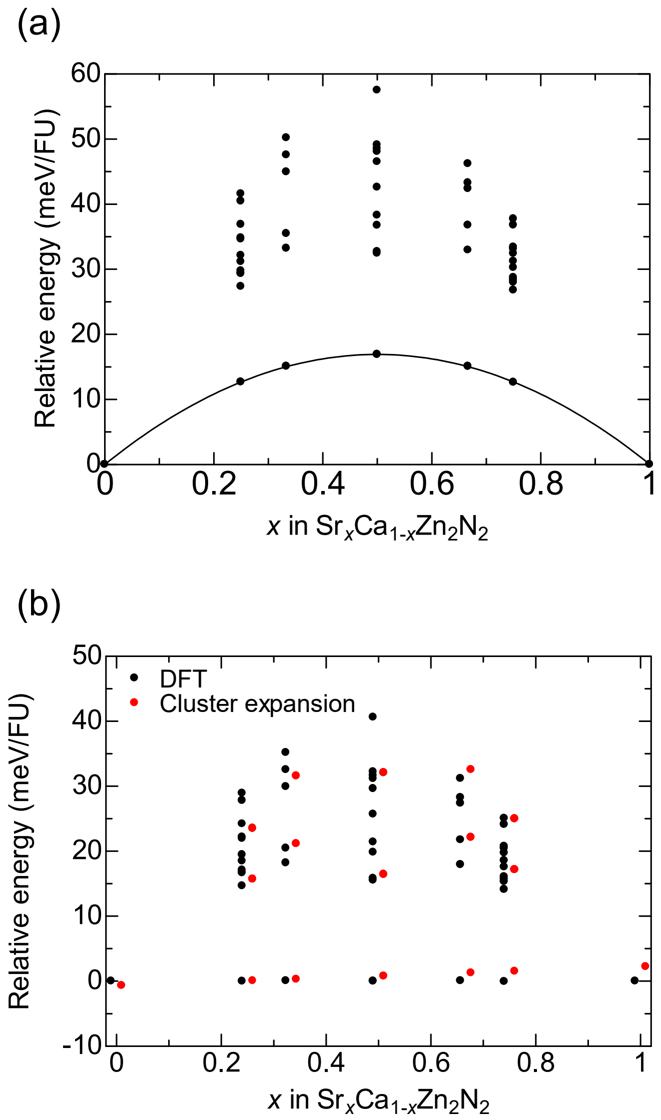

Supplementary Figure 17. DFT (PBE) and cluster expansion energies for $Sr_xCa_{1-x}Zn_2N_2$. (a) "Raw" DFT energies. (b) Comparison of DFT and cluster expansion energies. The quadratic energy correction of $67.6x(1-x)$ obtained from the fit in (a) is removed before fitting to a cluster expansion. Points are shifted in the horizontal direction for clarity.

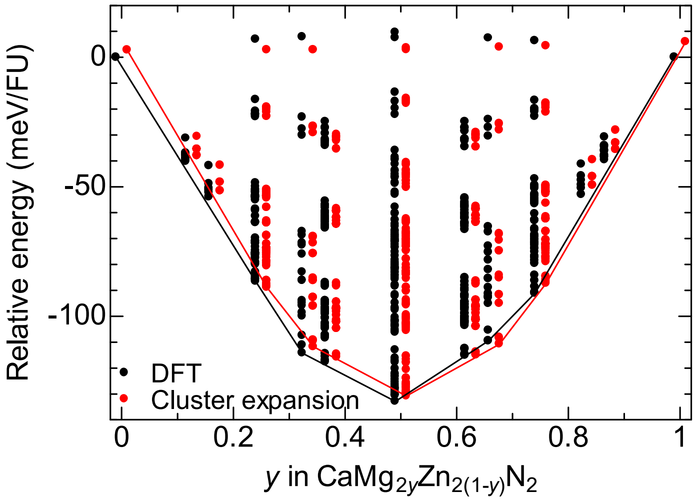

Supplementary Figure 18. DFT (PBE) and cluster expansion energies for $CaMg_{2y}Zn_{2(1-y)}N_2$. Points are shifted in the horizontal direction for clarity.

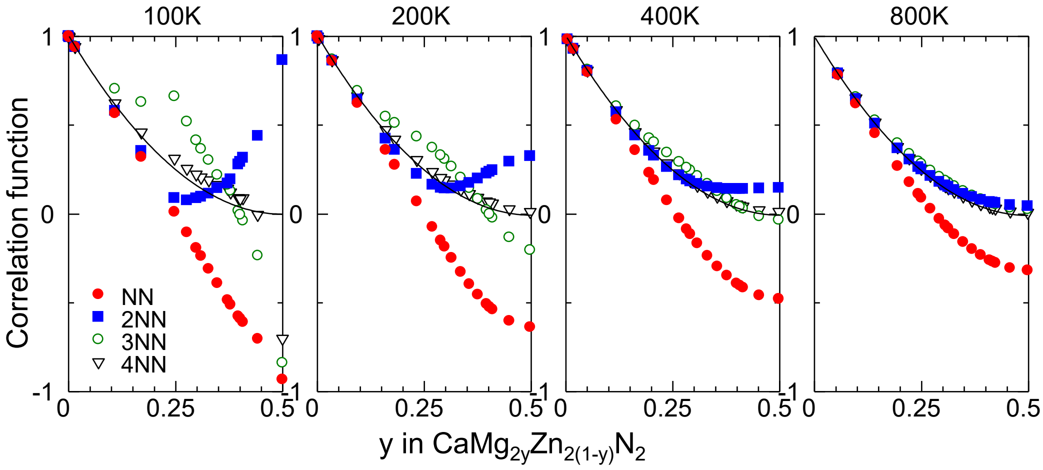

Supplementary Figure 19. First to fourth nearest neighbor (NN) correlation functions at various concentrations and temperatures in $CaMg_{2y}Zn_{2(1-y)}N_2$. The solid curve denotes the complete disorder limit.

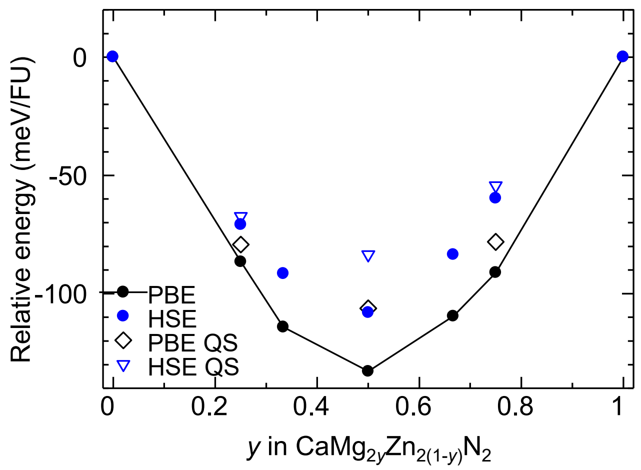

Supplementary Figure 20. Energies of PBE ground states and quasirandom structures as well as HSE06 energies of these structures in $CaMg_{2y}Zn_{2(1-y)}N_2$.

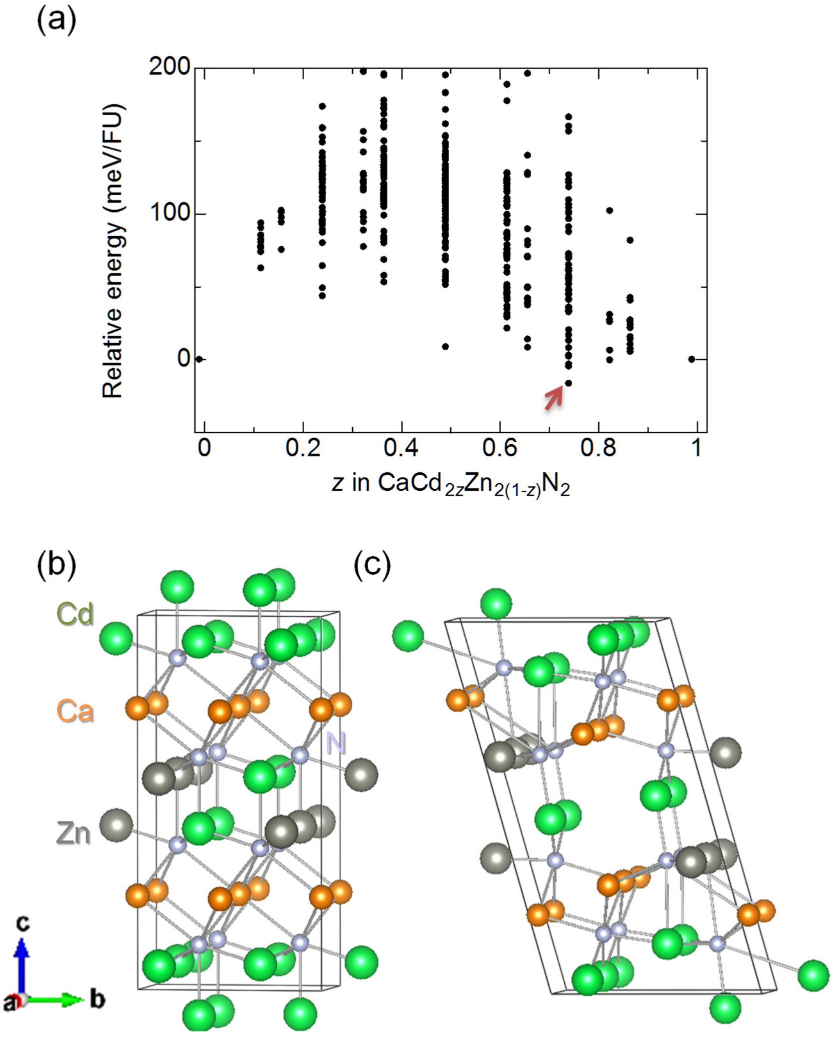

Supplementary Figure 21. Ordered structures for a cluster expansion of $CaCd_{2z}Zn_{2(1-z)}N_2$. (a) DFT (PBE) energies. (b) Lattice model and (c) a relaxed unit cell of the ground state $CaCd_{1.5}Zn_{0.5}N_2$ structure with a relative energy indicated by the red arrow in (a). The VESTA code[1] was used for illustration.

Supplementary Table 1 (pages 34 and 35). Prototype crystal structures considered in this study. The crystal structures for ternary nitrides that contain divalent cation sites are taken from the ICSD, where the divalent cation sites are to be occupied by Zn ions. The KCuO structure (*I*4*/mmm*), which has been theoretically predicted previously for NaZnN, KZnN, and RbZnN (ref. 7), is also considered for nitrides with a monovalent cation.

Prototype	Space group type	Space group number	Valence of cation M	ICSD number
LiZnN	$F\bar{4}3m$	216	1	16790
LiNiN	$P\bar{6}m2$	187	1	411151
NaSnN	$P6_3mc$	186	1	172471
LiSrN	$P4_2/mmc$	131	1	87414
LiCaN	*Pnma*	62	1	107304
LiBeN	$P12_1/c1$	14	1	402341
Li_4SrN_2	$I4_1/amd$	141	1	87413
KCuO	$I\bar{4}$	82	1	25695
CdC_2N_2	$P\bar{4}3m$	215	2	66938
$CaMg_2N_2$	$P\bar{3}m1$	164	2	411175
$BaBe_2N_2$	$I4/mcm$	140	2	415304
Ba_2ZnN_2	$I4/mmm$	139	2	80377
$CaGe_2N_2$	$P4_2/mbc$	135	2	280252
C_2HgN_2	$I\bar{4}2d$	122	2	15896
$GeSr_2N_2$	*Cmca*	64	2	153302
$NiSr_2N_2$	*Pnma*	62	2	91273
PbC_8N_6	$P31m$	157	2	171702
Mg_3BN_3	$P6_3/mmc$	194	3	79623
Sr_3GaN_3	$P6_3/m$	176	3	281259
C_3PN_3	$I\bar{4}2d$	122	3	16587
Ca_3AlN_3	$P12_1/c1$	14	3	410579
$Ba_3Al_2N_4$	*Pnna*	52	3	410578
$Sr_3Ga_2N_4$	*Pnna*	52	3	170441
$Ba_3B_2N_4$	$P2_12_12_1$	19	3	412663
$Ca_3Ga_2N_4$	$C12/c1$	15	3	170442

Supplementary Table 1. (*Continued.*)

Prototype	Space group type	Space group number	Valence of cation M	ICSD number
$Ca_3Al_2N_4$	$P12_1/c1$	14	3	280347
Sr_6GaN_5	$P6_3/mcm$	193	3	281260
$BaCeN_2$	$P6_3/mmc$	194	4	74792
$NiCN_2$	$P6_3/mmc$	194	4	249388
$BaCN_2$	$R\bar{3}c$	167	4	75041
$CaCN_2$	$R\bar{3}m$	166	4	418451
$SrCeN_2$	$R\bar{3}m$	166	4	95805
$BaZrN_2$	$P4/nmm$	129	4	74905
$CaGeN_2$	$I\bar{4}2d$	122	4	23523
$BaSiN_2$	*Cmca*	64	4	170268
$SrCN_2$	*Pnma*	62	4	75040
$CaSiN_2$	*Pbca*	61	4	170267
$PbCN_2$	$Pna2_1$	33	4	16600
$ZnSiN_2$	$Pna2_1$	33	4	656276
$HgCN_2$	$P12_1/a1$	14	4	412278
$SrSiN_2$	$P12_1/c1$	14	4	170266
Ca_4CN_4	*Pnma*	62	4	167787
Sr_4CN_4	*Pnma*	62	4	170798
Ca_4GeN_4	$P12_1/c1$	14	4	280251
$Sr_5Ge_2N_6$	$C12/c1$	15	4	419164
Sr_7GeN_6	*Pbcn*	60	4	249784
$Ba_2Si_5N_8$	$Pmn2_1$	31	4	401501
Ca_2PN_3	*Cmca*	64	5	72532
Zn_2PN_3	$Cmc2_1$	36	5	422150
Ca_2VN_3	$C12/c1$	15	5	409644
Sr_5NbN_5	*Pbcm*	57	5	412060
Ba_3MoN_4	*Pbca*	61	6	69689

Supplementary Table 2. Cation species considered in this study.

Valence	Elements
+1	Li, Na, K, Rb, Cs, Cu, Ag, Au, Tl
+2	Be, Mg, Ca, Sr, Ba, Cd, Hg, Si, Ge, Sn, Pb
+3	Sc, Y, La, B, Al, Ga, In, Tl, As, Sb, Bi
+4	Ti, Zr, Hf, Ce, Si, Ge, Sn, Pb
+5	V, Nb, Ta, P, As, Sb, Bi
+6	Cr, Mo, W, S, Se, Te

Supplementary Table 3. Crystal structures of the 21 identified nitrides. Those indicated with a hash symbol have relaxed into higher symmetry than the initial prototypes, and those with an asterisk are predicted using the evolutionary algorithm. The lattice parameters are for conventional cells determined using HSE06. Be_2ZnN_2 and Zn_3LaN_3 have relaxed significantly from the $NiSr_2N_2$ and Sr_3GaN_3 prototype structures, respectively [Supplementary Figs. 1(d) and (f)]. Crystal structures other than those in the prototype list (Supplementary Table 1) have been obtained for $ZnZrN_2$, $ZnHfN_2$, and Zn_3WN_4 using the evolutionary algorithm [see also Supplementary Figs. 1(i) and (l)]. The structure of Mg_2ZnN_2 predicted by the evolutionary algorithm is the same as the structure of Be_2ZnN_2 relaxed from the $NiSr_2N_2$ structure.

Chemical composition	Prototype	Space group type	Lattice parameters (Å) *a*	*b*	*c*
LiZnN	LiZnN	$F\bar{4}3m$	4.879		
NaZnN	KCuO #	*I*4/*mmm*	9.299		4.769
KZnN	KCuO #	*I*4/*mmm*	9.861		5.761
$CaZn_2N_2$	$CaMg_2N_2$	$P\bar{3}m1$	3.454		5.990
Be_2ZnN_2	$NiSr_2N_2$ #	*I*4/*mmm*	3.109		10.452
Mg_2ZnN_2	Be_2ZnN_2*	*I*4/*mmm*	3.911		10.449
Ca_2ZnN_2	Ba_2ZnN_2	*I*4/*mmm*	3.575		12.607
Sr_2ZnN_2	Ba_2ZnN_2	*I*4/*mmm*	3.853		12.880
Ba_2ZnN_2	Ba_2ZnN_2	*I*4/*mmm*	4.184		13.054
Zn_3LaN_3	Sr_3GaN_3	$P6_3/m$	8.539		3.308
$ZnSiN_2$	$ZnSiN_2$	$Pna2_1$	5.240	6.268	5.022
$ZnGeN_2$	$ZnSiN_2$	$Pna2_1$	5.472	6.456	5.202
$ZnSnN_2$	$ZnSiN_2$	$Pna2_1$	5.854	6.749	5.468
$ZnTiN_2$	$ZnSiN_2$	$Pna2_1$	5.674	6.557	5.207
$ZnZrN_2$	$ZnZrN_2$*	$P3m1$	3.274		5.292
$ZnHfN_2$	$ZnZrN_2$*	$P3m1$	3.237		5.294
Zn_2PN_3	Zn_2PN_3	$Cmc2_1$	9.360	5.463	4.921
Zn_2VN_3	Zn_2PN_3	$Cmc2_1$	9.513	5.584	5.238
Zn_2NbN_3	Zn_2PN_3	$Cmc2_1$	9.852	5.782	5.386
Zn_2TaN_3	Zn_2PN_3	$Cmc2_1$	9.856	5.765	5.387
Zn_3WN_4	Zn_3WN_4*	$Pmn2_1$	6.548	5.666	5.328

Supplementary Table 4 (pages 38 and 39). Band gaps of the identified nitrides and their relevant nitrides. Theoretical minimum (M) and direct (D) band gaps are compared with experimental values (units in eV). The M and D values are equal in a direct-type band structure and different in an indirect-type band structure. GW_0@HSE06 is used instead of GW_0@PBE for $CaCd_2N_2$ and InN (indicated with asterisks) because no band gap with PBE would yield inaccurate W_0. GW_0 calculations were not performed for Zn_3N_2, Mg_3N_2, and Ca_3N_2 due to large unit cells. The actual minimum band gaps are smaller when the band edges are not located at the sampled *k*-points. This applies to NaZnN, Be_2ZnN_2, and $ZnTiN_2$, for which the actual minimum indirect band gaps estimated from the band diagrams using HSE06 [Fig. 1(a) in the main article and Supplementary Fig. 4] are smaller than the tabulated values by 0.05, 0.25, and 0.04 eV, respectively.

	HSE06		GW_0@PBE		Experiment		
	M	D	M	D			
LiZnN	1.84	1.84	1.74	1.74	1.91 [8]		
NaZnN	1.08	1.85	1.40	2.14			
KZnN	1.22	1.38	1.60	1.77			
$CaZn_2N_2$	1.83	1.83	1.79	1.79	1.9 [a]		
Be_2ZnN_2	3.23	4.03	3.65	4.45			
Mg_2ZnN_2	2.49	3.22	3.05	3.78			
Ca_2ZnN_2	1.65	1.92	1.72	1.86	1.6 (M) [a]	1.9 (D) [a]	
Sr_2ZnN_2	1.64	1.64	1.75	1.75			
Ba_2ZnN_2	1.27	1.40	1.47	1.57			
Zn_3LaN_3	1.73	1.73	2.00	2.00			
$ZnSiN_2$	4.81	5.16	5.17	5.53			
$ZnGeN_2$	3.18	3.18	3.38	3.38	3.2 [9]		
$ZnSnN_2$	1.39	1.39	1.41	1.41	1.0 [10]	1.7 [11]	2.0 [9]
$ZnTiN_2$	3.45	3.85	4.68	4.72			
$ZnZrN_2$	3.25	3.94	3.27	3.69			
$ZnHfN_2$	3.39	3.73	3.46	3.59			
Zn_2PN_3	4.14	4.97	4.40	5.25			
Zn_2VN_3	2.30	2.48	3.29	3.48			
Zn_2NbN_3	3.04	3.09	3.46	3.51			
Zn_2TaN_3	3.52	3.58	3.65	3.93			
Zn_3WN_4	3.80	4.01	3.84	3.96			

[a] Experimental values obtained in this work.

Supplementary Table 4. (*Continued.*)

	HSE06		GW_0@PBE		Experiment
	M	D	M	D	
$CaMg_2N_2$	3.26	3.26	3.69	3.69	3.20 [b]
$CaCd_2N_2$	0.44	0.44	0.51*	0.51*	
$SrZn_2N_2$	1.60	1.60	1.50	1.50	
Zn_3N_2	0.84	0.84	-	-	1.06 [12]
Mg_3N_2	2.77	2.77	-	-	2.75 [b]
Ca_3N_2	2.03	2.07	-	-	1.70 [b]
GaN	3.10	3.10	3.21	3.21	3.39 [13]
InN	0.63	0.63	0.72*	0.72*	0.67 [14]

[b] Values estimated by the present authors from absorption spectra reported in ref. 15 using a plot of $(h\nu\alpha/S)^2$ vs. $h\nu$.

Supplementary Table 5 (pages 40 and 41). Effective masses for holes and electrons in units of the free electron rest mass and the considered directions for relevant compounds. Points in the Brillouin zone are labeled based on the standard cell defined by Setyawan and Curtarolo[3]. The HSE06 results for the directions that provide the smallest effective masses are shown. Heavy holes are considered when the VBM is degenerate because of their dominant contributions to the DOS near the VBM. Similarly, heavy-hole-like bands are sampled when the VBM is nearly degenerate (less than 0.1 eV differences in band energies). This treatment is used for $ZnGeN_2$, $ZnSnN_2$, and $CaMg_2N_2$ to avoid sampling of split-off-like bands, which have much smaller effective masses and therefore much less contribute to the DOS. The values in parentheses indicate the actual smallest hole effective masses of such split-off-like bands. An asterisk denotes that a single, non-degenerate band exists at the VBM. The electron effective masses for $ZnZrN_2$ and $ZnHfN_2$ are not shown because their lowest conduction bands are too flat for accurate quadratic fitting.

	Hole		Electron	
	Effective mass	Direction	Effective mass	Direction
LiZnN	0.74	Γ-X	0.18	Γ-K, Γ-L, Γ-X
NaZnN	1.00*	M-Z_1	0.27	Γ-M, Γ-X
KZnN	1.22*	M-Z_1	0.33	Γ-M, Γ-X
$CaZn_2N_2$	0.91	Γ-K, Γ-M	0.17	Γ-A, Γ-K, Γ-M
Be_2ZnN_2	1.12	Z-Y_1	0.39	Near Σ_1 on Σ_1-Z
Mg_2ZnN_2	0.98	Z-Γ	0.34	Γ-N, Γ-X, Γ-Σ
Ca_2ZnN_2	1.00	Z-Σ_1	0.54	Near Σ on Σ-Γ
Sr_2ZnN_2	0.85	Γ-X	0.21	Γ-Z
Ba_2ZnN_2	0.68	Γ-Σ	0.35	X-P
Zn_3LaN_3	0.20*	Γ-M	0.30	Γ-K, Γ-M
$ZnSiN_2$	0.52	U-Z	0.33	Γ-X, Γ-Z
$ZnGeN_2$	2.06	Γ-Y	0.20	Γ-X, Γ-Y
	(0.20	Γ-Y)		
$ZnSnN_2$	1.96	Γ-Y	0.11	Γ-X, Γ-Y, Γ-Z
	(0.12	Γ-Z)		
$ZnTiN_2$	1.83	U-Z	1.11	Between Γ-Y
$ZnZrN_2$	0.67	Γ-K, Γ-M	Large	Between L-M
$ZnHfN_2$	0.67	Γ-K, Γ-M	Large	Between L-A

Supplementary Table 5. (*Continued.*)

	Hole		Electron	
	Effective mass	Direction	Effective mass	Direction
Zn_2PN_3	0.59*	X-Γ, X-S	0.32	Γ-X, Γ-Z
Zn_2VN_3	1.37*	Near X on X-Γ	0.59	Y-T
Zn_2NbN_3	0.99*	X-S	0.39	Y-T
Zn_2TaN_3	1.01*	X-S	0.43	Y-T
Zn_3WN_4	1.00*	Y-Γ	0.57	Γ-Z
$CaMg_2N_2$	1.79	Γ-K, Γ-M	0.20	Γ-A
	(0.20	Γ-A)		
$CaCd_2N_2$	1.00	Γ-K, Γ-M	0.08	Γ-K, Γ-M
$SrZn_2N_2$	0.95	Γ-K, Γ-M	0.15	Γ-A, Γ-K, Γ-M
Zn_3N_2	0.99	Γ-H	0.08	Γ-H, Γ-N, Γ-P
GaN	1.97	Γ-A	0.18	Γ-A

Supplementary Table 6. Lattice parameters of Ca_2ZnN_2 and $CaZn_2N_2$ determined by the Pawley method and reliability factors (*R*) obtained from Rietveld analysis. Values in parentheses are standard deviations in the last digit.

	Ca_2ZnN_2	$CaZn_2N_2$
a (Å)	3.583926(70)	3.46380(11)
c (Å)	12.66449(27)	6.00969(30)
R_{wp} (%)	4.15	7.81
R_{exp} (%)	3.48	4.86
GOF	1.19	1.61
R_{Bragg} (%)	0.450	4.96

Supplementary Table 7. Structure parameters of Ca_2ZnN_2 and $CaZn_2N_2$. Multiplicities and Wyckoff notations (WN), site occupancies (*g*), fractional coordinates (*x*, *y*, *z*), and isotropic displacement parameters (*B*) refined by the Rietveld method are shown. Values in parentheses are standard deviations in the last digit.

Formula and site	WN	*g*	*x*	*y*	*z*	*B* (Å^2)
Ca_2ZnN_2						
Ca	4*e*	1.0	0	0	0.33550(25)	0.462(54)
Zn	2*a*	1.0	0	0	0	0.361(75)
N	4*e*	1.0	0	0	0.15221(68)	1.26(22)
$CaZn_2N_2$						
Ca	1*b*	1.0	0	0	1/2	0.95(11)
Zn	2*d*	1.0	1/3	2/3	0.86232(32)	0.664(39)
N	2*d*	1.0	1/3	2/3	0.2390(15)	0.30(23)

Supplementary Table 8. Lattice parameters for the conventional cells of derivative compounds of $CaZn_2N_2$ determined using HSE06. Mismatches versus $CaZn_2N_2$ are also shown. $CaMg_2N_2$, $CaCd_2N_2$, $SrZn_2N_2$, and $BaZn_2N_2$, which are isostructural to $CaZn_2N_2$, are considered as candidates of alloying agents. Among these, only $CaMg_2N_2$ has been reported experimentally. Our calculations indicate that $CaCd_2N_2$ and $SrZn_2N_2$ are dynamically stable (Supplementary Fig. 2). $SrZn_2N_2$ is metastable against competing phases according to the phase diagram calculations (Supplementary Fig. 3), while the phase diagram of the Ca-Cd-N system has not been investigated. $BaZn_2N_2$ is found to be dynamically unstable (Supplementary Fig. 2) and excluded from consideration as an alloy agent.

Chemical composition	Lattice parameters (Å)		Mismatch vs $CaZn_2N_2$	
	a	*c*	*a*	*c*
$CaZn_2N_2$	3.454	5.990	-	-
$CaMg_2N_2$	3.528	6.047	2.1%	1.0%
$CaCd_2N_2$	3.695	6.526	7.0%	8.9%
$SrZn_2N_2$	3.539	6.236	2.5%	4.1%

Supplementary Note 1: Nitrogen chemical potential conditions for the formation of $CaZn_2N_2$

Figure 2(a) in the main article shows that the lower limit of the N chemical potential in the $CaZn_2N_2$ phase region is given by condition IV, where $\Delta\mu_N = -0.30$ eV. To form the $CaZn_2N_2$ phase, the N chemical potential is required to be higher than this value. Although the phase diagram has been constructed at 0 K and 0 GPa, the temperature and pressure dependence of phase stability can be approximately discussed via considering the temperature and partial pressure dependence of the N_2 gas phase that has a much stronger dependence than the solid phases. Supplementary Figure 6 shows the dependence of the N chemical potential on temperature and N_2 partial pressure using the ideal gas model[4] in conjunction with a fugacity coefficient estimated at high temperature and pressure[5]. At temperatures higher than ~450 K, $\Delta\mu_N = -0.30$ eV corresponds to a N_2 partial pressure of higher than 0.1 MPa. A partial pressure in a gigapascal order would be required at temperatures higher than 800 K. We therefore opted for high-pressure synthesis of $CaZn_2N_2$. Indeed, we found that a conventional approach used for the synthesis of Ca_2ZnN_2 cannot form $CaZn_2N_2$ as described in the main article.

Supplementary Figure 7 shows a Ca-Zn-N ternary chemical potential diagram at 0 K and 5 GPa. The lower limit of the N chemical potential in the $CaZn_2N_2$ phase region is $\Delta\mu_N = -0.30$ eV, which is almost the same as that at 0 GPa. Supplementary Figure 6 indicates that temperatures lower than 1400 K are required to form $CaZn_2N_2$ at 5 GPa. Our experimental conditions of 1473 K and 5 GPa are reasonably close to the theoretically estimated stable region of $CaZn_2N_2$.

Supplementary Note 2: Theoretical band gaps

The band gap calculated using the HSE06 hybrid functional and the *GW* approximation are summarized in Supplementary Table 4. The *GW* calculations were performed on top of the PBE-GGA (GW_0@PBE), where the quasi-particle energies in the Green's function *G* were iteratively updated to self-consistency and the screened Coulomb interaction W_0 was obtained using the random phase approximation without update. This has been shown to give a good screening property similar to self-consistent, vertex corrected *W* (ref. 16). Exceptions are $CaCd_2N_2$ and InN, for which metallic band structures with PBE would result in inaccurate W_0 and, therefore, GW_0 calculations were performed on top of HSE06 (GW_0@HSE06). Both GW_0@PBE and GW_0@HSE06 calculations were conducted using HSE06 lattice parameters because HSE06 typically yields the lattice parameters of semiconductors closer to experimental values compared to PBE. *GW* calculations have not been carried out for Zn_3N_2, Mg_3N_2, and Ca_3N_2 due to the large unit cell of the bixbyite structure (containing 40 atoms in the primitive cell).

The band gaps from HSE06 and GW_0 calculations are similar for many of the nitrides considered here. Exceptions are $ZnTiN_2$ and Zn_2VN_3, for which GW_0 values are ~1 eV larger. Although the performance of *GW* has not been well established for $3d^0$ transition-metal nitrides, it has been reported that *GW* using the random phase approximation leads to band gap overestimations by ~1 eV or larger for $3d^0$ transition-metal oxides such as TiO_2 (refs. 17,18), $SrTiO_3$ (ref. 18), and V_2O_5 (ref. 17). Vertex corrections in the self-energy are likely to improve the band gaps via shifting the 3*d* states[19], but such calculations are computationally demanding and have not been reported yet for $3d^0$ transition-metal compounds. In addition, contributions of lattice polarization to the screening can significantly reduce the band gaps[20-22]. Considering these known issues for oxides, we assume that our GW_0 band gaps are also overestimated for $ZnTiN_2$ and Zn_2VN_3.

When compared with available experimental values, HSE06 shows generally good agreements with experiments and appears to perform slightly better than GW_0. In particular, a sizable overestimation by GW_0 is found for $CaMg_2N_2$. For these reasons, we use the results of HSE06 calculations in the present study.

Supplementary Method 1: Screening procedure

We initiate our search for new semiconductors by obtaining prototypes of crystal structures. Compounds containing a divalent cation, another cation, and N^{3-} are extracted from the Inorganic Crystal Structure Database (ICSD)[23,24]; those with any sites with fractional occupancies or any cations not shown in Supplementary Table 2 are not considered. The list of 52 prototypes considered in this study is provided as Supplementary Table 1. Initial structures of individual systems are obtained by replacing the divalent cation with Zn and the other cation with a cation listed in Supplementary Table 2 with the same valence (denoted as M hereafter). Neither the prototypes nor the substituting ions exclude non-earth-abundant elements because the electronic structure of compounds involving any element can provide useful information on the design and search of novel semiconductors. For systems with two kinds of divalent cations, Zn could either be the first or second cation. This yields 583 ternary nitrides to be investigated, which consist of various polymorphs of 125 chemical formulae.

In view of the feasibility of experimental synthesis, semiconductors of interest should be dynamically stable, that is, contain no imaginary phonon modes, and also not be too unstable thermodynamically. The following procedure is used in the computational screening. The order of the screening steps is mostly based on the computational cost: less expensive calculations are employed at more initial stages where more candidates need to be considered.

1) The lattice parameters and internal coordinates are relaxed using the PBE-GGA for the 583 ternary nitrides. The *k*-point meshes are determined on the basis of the convergence of total energies as mentioned in the Methods section in the main article.

2) Calculations based on density functional perturbation theory (DFPT) are conducted using the PBE-GGA to identify whether there are any imaginary phonon modes at the Γ point. If a compound is dynamically unstable at the Γ point, which means that there is at least one imaginary mode, the compound is not considered in the following steps.

3) A preliminary check of thermodynamic stability is carried out using the PBE-GGA. Here, the formation energy of an $M_lZn_mN_n$ ternary nitride is compared against pseudo-binary end members M_3N_v and Zn_3N_2, where v is the valence of M. When M_3N_v is unstable, an energy after decomposition into simple substances and/or binary phases other than M_3N_v is used: for instance, $3M + v/2\ N_2$ when there are no stable M-N

compounds. The formation energy of 3Zn + N_2 is used for Zn_3N_2 because Zn_3N_2 is unstable against 3Zn + N_2 with the PBE-GGA by 0.11 eV/atom (formation energy at 0 K), while experimental formation enthalpy of Zn_3N_2 at 298 K is -0.05 eV/atom (ref. 25). The ~0.15 eV/atom differences between theoretical formation energies and experimental formation enthalpies are also found in Mg_3N_2 and Ca_3N_2: the theoretical formation energy of Mg_3N_2 is -0.78 eV/atom, compared to an experimental value of -0.96 eV/atom (ref. 25), and the theoretical formation energy of Ca_3N_2 is -0.81 eV/atom, compared to an experimental value of -0.91 meV/atom (ref. 25). To partly accommodate errors associated with the PBE-GGA and the omission of temperature effects, stable and slightly metastable compounds with formation energies less than 0.05 eV/atom are subject to further investigation.

4) Further screening for thermodynamic stability is carried out by deriving ternary phase diagrams using the PBE-GGA. Competing phases that are reported to be stable in the Materials Project database[26] are considered, and the $CaZn_{13}$ phase[27] that was occasionally found in our experiment is additionally included for the Ca-Zn-N ternary system. The main objective is to exclude systems that are actually unstable against competing phases but was not excluded in step 3. Again, compounds that are thermodynamically metastable but with the energies less than 0.05 eV/atom of the convex hull are not excluded from consideration.

5) The crystal structures of remaining compounds are also predicted using an evolutionary algorithm. This was performed in order to validate the aforementioned structure search within the crystal structures reported in the ICSD without bias towards previously reported structures. The evolutionary algorithm as implemented in the USPEX code[28] is used in conjunction with PBE-GGA calculations. The crystal structures with the lowest total energy, whether that be from the ICSD or evolutionary algorithm screening, is considered to be the ground state for each case.

Ideally, the evolutionary algorithm crystal structure predictions should be performed for all initial candidates in step 1 but it is computationally too demanding: one structural search using the evolutionary algorithm typically requires first-principles calculations for ~500 to ~1000 structures. We therefore performed the crystal structure predictions for only those systems screened to the shortlist from step 4 and only if they are unreported previously.

6) The phonon density of states is calculated using the finite displacement method as implemented in the Phonopy code[29] and the PBE-GGA. Systems with imaginary phonon modes are excluded from candidates.

7) Metallic systems are identified via band diagram calculations using the HSE06 hybrid functional after geometry optimization and then removed. However, all of the nitrides that remain up to step 6 have non-zero band gaps and, therefore, no system has been removed from the candidates at this step.

8) Systems with small effective masses are selected using the band structures obtained using the HSE06 hybrid functional. The criteria are hole and electron effective masses smaller than $2m_0$, where m_0 is the free electron rest mass. Heavy holes are considered when the VBM is degenerate as they contribute mainly to the DOS near the VBM. Similarly, heavy-hole-like bands are sampled when the VBM is nearly degenerate (less than 0.1 eV differences in band energies). This step has not screened out any systems since all the remaining met the criteria: either or both of hole and electron effective masses are smaller than $2m_0$.

Supplementary Method 2: Compilation of pseudo-binary phase diagrams

The band gap of $CaZn_2N_2$ is expected to be tunable by substituting Sr for Ca or Mg for Zn. To understand whether $Sr_xCa_{1-x}Zn_2N_2$ and $CaMg_{2y}Zn_{2(1-y)}N_2$ alloys form at a given temperature, pseudo-binary phase diagrams are compiled through the cluster expansion[30-32] and Monte Carlo simulations. The cluster expansion technique employs a lattice model and identifies sites that can be occupied by multiple species. The species at lattice site *i* is distinguished using a pseudo-spin configurational variable σ_i, and the objective is to expand the energy of the system using these variables. Within the binary cluster expansion formalism, the configurational energy from the cluster expansion E_{CE} can be expressed using effective cluster interactions (ECIs) as

$$E_{\mathrm{CE}} = V_0 + \sum_i V_i \sigma_i + \sum_{i,j} V_{ij} \sigma_i \sigma_j + \cdots = \sum_\alpha V_\alpha \varphi_\alpha$$

where α represents clusters (examples are the point cluster and the nearest neighbor pair cluster), V_α is the ECI for cluster α, and φ_α is the correlation function for cluster α. The correlation function is taken to be between -1 and 1 when the configurational variables are -1 and 1.

A suitable cluster expansion should reasonably reproduce the density functional theory (DFT) energy E_{DFT} using a small set of clusters. The lattice parameters and internal coordinates are allowed to relax in DFT calculations. The values of ECIs are determined for the set of adopted clusters through a least-squares fit that minimizes the sum of squared errors between E_{CE} and E_{DFT} for all structures in the training set. The choice of which clusters, that is, interactions between lattice sites, to include determines the goodness of the cluster expansion. The set of clusters that minimizes the leave-one-out cross-validation (CV) score [33,34] is often used. Here, overfitting should be avoided, especially for systems where using a very small number of clusters is appropriate. The selected clusters and derived ECIs should reflect the interactions in the system. Moreover, compositions where ground state structures, or structures on the convex hull, appear should ideally be the same in DFT and cluster expansion.

Grand canonical Monte Carlo simulations are used to obtain a pseudo-binary phase diagram. If one sweeps the chemical potential continuously and monotonously at a fixed temperature, the concentration would continuously and monotonously change in a solid solution region but would abruptly jump at a critical chemical potential corresponding to the boundaries of a two-phase region. There could be many two-phase regions at 0 K because concentrations that are not on the convex hull lie in a two-phase region. Critical

chemical potentials corresponding to the phase boundaries at 0 K can be analytically obtained from slopes on the E_{CE}-concentration plot. In this study, a $12\times12\times12$ formula unit (FU) supercell is used for Monte Carlo simulations, and averaging is carried out in each Monte Carlo simulation temperature over 20,000 steps/site after discarding 10,000 steps/site for equilibration. One FU is equal to one primitive cell in the systems discussed in this section.

$Sr_xCa_{1-x}Zn_2N_2$. Supplementary Figure 16 shows the crystal structure of $CaZn_2N_2$. Ca/Sr sites form layers of triangular lattices. The PBE-GGA functional is used to obtain DFT energies of all possible 50 Ca/Sr configurations in supercells consisting of up to four FUs. Supplementary Figure 17(a) shows the DFT energies relative to the two end members ($CaZn_2N_2$ and $SrZn_2N_2$). A peculiar characteristic is that the energies of structures with relatively low energies can be fitted to a parabola where this quadratic energy contribution is proportional to $x(1-x)$. The structures on this parabola are lamellar with layers of either Sr or Ca stacking along the axial direction.

Supplementary Figure 17(b) shows the DFT and cluster expansion energies of structures in the training set. Here, the quadratic energy correction of $67.6x(1-x)$ meV/FU from the fit in Supplementary Fig. 17(a) is removed from the energies calculated with DFT. This correction models the strain energy due to forcing the in-plane lattice parameters of $CaZn_2N_2$ and $SrZn_2N_2$ layers to be the same. The cluster expansion formalism does not, in principle, require this correction. However, the interaction between layers of $CaZn_2N_2$ and $SrZn_2N_2$ is long-ranged and too many clusters would be required to represent this interaction. Therefore, the quadratic correction is applied to obtain a cluster expansion with as few clusters as possible. The configurational variable is set to -1 if the corresponding (Sr/Ca) site is occupied by Sr and 1 if by Ca. The empty, point, and nearest neighbor pair clusters are considered. The negative ECI for the pair cluster, which is -23.5 meV/FU, means that like species attract each other. The CV score and root-mean-square average error are 0.5 and 3.2 meV/FU, respectively.

We are interested in the solubility limit of Sr in $Sr_xCa_{1-x}Zn_2N_2$. The boundary can be approximated using a standard cluster expansion through the following procedure as long as x at the two-phase boundary of $Sr_xCa_{1-x}Zn_2N_2$ does not deviate much from 0 or 1. The cluster expansion energy per Monte Carlo supercell consisting of N sites in the considered system is

$$E_{CE} = NV_0 + V_1\sum_i \sigma_i + V_2\sum_{ij} \sigma_i\sigma_j + NV_3x(1-x).$$

Here, V_0 , V_1, and V_2 are ECIs for empty, point, and nearest neighbor pairs divided by multiplicity (1, 1, and 6, respectively), V_3 is the quadratic correction term (V_3 = 67.6 meV/FU), and the concentration x is equivalent to

$$x = \left(N - \sum_i \sigma_i \right) \Big/ 2N .$$

The summation for i is over all sites and that for i and j is over all nearest neighbor pair clusters in the Monte Carlo supercell. The Hamiltonian in a grand canonical Monte Carlo simulation is the grand potential Ω that can be written as

$$\Omega = E_{CE} - \mu \sum_i \sigma_i = NV_0 + (-\mu + V_1) \sum_i \sigma_i + V_2 \sum_{ij} \sigma_i \sigma_j + NV_3 x(1-x).$$

The absolute value of the grand potential is irrelevant as only relative values are meaningful for compilation of the phase diagram; therefore, the first term can be removed. Redefining the chemical potential as $\mu' = \mu - V_1$, the grand potential

$$\Omega' = -\mu' \sum_i \sigma_i + V_2 \sum_{ij} \sigma_i \sigma_j + NV_3 x(1-x)$$

is considered hereon. Let us first consider the situation when the third term is absent:

$$\Omega'_0 = -\mu' \sum_i \sigma_i + V_2 \sum_{ij} \sigma_i \sigma_j$$

with a repulsive pair ECI $V_2 < 0$. There would be two ground states at 0 K, which are the two end members $\sum_i \sigma_i = N \quad (x = 0)$ and $\sum_i \sigma_i = -N \quad (x = 1)$. The critical chemical potential is 0 from symmetry, and the former and latter end members correspond to $\mu' > 0$ and, $\mu' < 0$ respectively.

Now the third term is included. We consider very small deviations in the concentration from either 0 or 1, which is the limit at low temperature. The second-order term of x is removed using the approximation $x(1-x) \approx x \ (x \approx 0)$ or $x(1-x) \approx 1 - x \ (x \approx 1)$. The two approximations to Ω' are

$$\Omega'_1 = -\mu' \sum_i \sigma_i + V_2 \sum_{ij} \sigma_i \sigma_j + V_3 \left(N - \sum_i \sigma_i \right) \Big/ 2 \quad \text{if } x \approx 0 \text{ hence } \mu' > 0$$

$$\Omega'_2 = -\mu' \sum_i \sigma_i + V_2 \sum_{ij} \sigma_i \sigma_j + V_3 \left(N + \sum_i \sigma_i \right) \Big/ 2 \quad \text{if } x \approx 1 \text{ hence } \mu' < 0$$

The former can be written as

$$\Omega_1' = V_3 N/2 - (\mu' + V_3/2)\sum_i \sigma_i + V_2 \sum_{ij} \sigma_i \sigma_j \quad \text{if} \quad x \approx 0 \quad \text{hence} \quad \mu' > 0$$

where the maximum x (≈ 0) that can be reached in equilibrium can be obtained by a grand canonical Monte Carlo simulation. The employed simulation is a fixed chemical potential standard cluster expansion with grand potential $\Omega'' = -\mu'' \sum_i \sigma_i + V_2 \sum_{ij} \sigma_i \sigma_j$. Using a chemical potential of $\mu'' = V_3/2$ results in the grand potential becoming $-(V_3/2)\sum_i \sigma_i + V_2 \sum_{ij} \sigma_i \sigma_j$, which is Ω_1' with $\mu' = 0$ and the constant term removed. A similar argument is possible for $x \approx 1$.

Supplementary Figure 12 shows the phase diagram for $Sr_xCa_{1-x}Zn_2N_2$. The two-phase boundaries can be derived by using two grand canonical Monte Carlo simulations with μ' slightly over 0 and another with μ' slightly under 0. At low temperature, the phase boundaries shown with solid curves are drawn along points from simulations at fixed chemical potentials very close to the critical chemical potential of $\mu' = 0$. However, at higher temperature where the simulated boundaries deviate much from 0 or 1, the approximations $x(1-x) \approx x$ ($x \approx 0$) or $x(1-x) \approx 1-x$ ($x \approx 1$) would not be appropriate and the actual boundary should be closer to x = 0.5. The quadratic energy correction $67.6x(1-x)$ becomes more overestimated as the deviation in x from 0 or 1 increases, which results in an extra driving force toward phase separation. Removing this driving force would narrow the two-phase coexistence region width. In consequence, the solid curves indicating the two-phase region boundaries are drawn up to ~500 K only. About 10% mixing is anticipated at 500 K, and at least 25% mixing would be tolerated at 800 K. Alloys are likely to be obtained for most compositions above ~1000 K.

To investigate the band gaps of $Sr_xCa_{1-x}Zn_2N_2$ alloys, a special quasirandom structure (SQS) [35,36] is employed, where φ_α is as close as possible to a random structure. For $Sr_xCa_{1-x}Zn_2N_2$, φ_α of a perfectly random structure is $(1-2x)/2$ for the point cluster and $(1-2x)^2$ for pair clusters. A SQS exists at x=0.5 where $\varphi_\alpha = 0$ for the five nearest neighbors. The PBE-GGA formation energy of the SQS with regard to the two end members is 40 meV/atom (23 meV/atom from the quadratic energy correction). An additional SQS is considered each at x = 0.25 and x = 0.75. These SQSs are used to evaluate the band gaps of $Sr_xCa_{1-x}Zn_2N_2$ alloys, as shown in Fig. 4(b) in the main article.

$CaMg_{2y}Zn_{2(1-y)}N_2$. As shown in Supplementary Fig. 16, Mg/Zn triangular lattice layers exist as pairs and appear as a honeycomb lattice when viewed perpendicular to the layers [Supplementary Fig. 16(b)]. Supplementary Figure 18 shows the DFT (PBE-GGA) and cluster expansion energies of structures in the training set. All possible 449 Mg/Zn configurations in supercells consisting of up to four FU are considered. Again, energies are relative to the two end members ($CaZn_2N_2$ and $CaMg_2N_2$). The configurational variable is set to -1 if the corresponding (Mg/Zn) site is occupied by Mg and 1 if by Zn. The empty, point, and up to sixth-nearest neighbor pair clusters are included in the $CaMg_{2y}Zn_{2(1-y)}N_2$ cluster expansion. The pair clusters are indicated in Supplementary Fig. 16. The ECIs V_α are 84.2, 14.8, -9.7, -1.4, -7.7, and 2.1 meV/FU for the first to sixth nearest neighbor pair clusters, respectively. The ECIs of the two closest pairs are largely positive, which indicates attraction between unlike species. The magnitudes of the pair clusters show a trend to decrease with increasing size, which suggests good convergence of the cluster expansion. The CV score and root-mean-square average errors are 2.1 and 2.2 meV/FU, respectively.

Supplementary Figure 11(a) shows the pseudo-binary phase diagram of $CaMg_{2y}Zn_{2(1-y)}N_2$. The points in the phase diagram are concentrations from fixed temperature simulations where the chemical potential is either gradually decreased (black crosses) or increased (red crosses). The blue curves, which indicate phase boundaries between single and two-phase regions, are estimated though concentration-chemical potential plots; examples of such plots are given in Supplementary Figs. 11(b) and (c). Two-phase regions cannot be accessed in equilibrium, and therefore the concentration should jump at a critical chemical potential when there is a two-phase region. However, in practice, there is hysteresis in the concentration-chemical plot that should be taken into account when determining boundaries, such as those found at 125 K in Supplementary Fig. 11(b). At 300 K the concentration-chemical potential is smooth and there is no hysteresis [Supplementary Fig. 11(c)], which suggests that there are no two-phase regions at 300 K. Therefore, $CaMg_2N_2$ and $CaZn_2N_2$ are expected to form a solid solution at any concentration at room temperature.

Supplementary Figure 19 shows how the correlation functions for the four nearest neighbor pairs change with temperature. Only the range $0 \le y \le 0.5$ is shown because of symmetry about $y = 0.5$. The correlation function converges gradually to the parabola (correlation function for a random structure) with large pair clusters converging faster. However, the correlation function for the nearest neighbor pair does not converge to the parabola at 800K. The nearest neighbor correlation function at $y = 0.5$ is roughly -1/3

even at 800K and does not approach 0. Recalling that there are three nearest neighbors for each Mg/Zn, the correlation of the pair cluster at y = 0.5 would be -1/3 if all Mg ions are nearest neighbors with two Zn ions and all Zn ions with two Mg ions. This type of very short range (nearest neighbor) ordering is expected to remain for y = 0.5 at 800K. For $CaMg_{2y}Zn_{2(1-y)}N_2$, this study investigates quasirandom structures (QSs) searched with φ_α as close to the random structure as possible for second to 11th nearest neighbors in supercells up to eight formula units instead of standard SQSs. We note that the distance between the Mg(Zn)-Mg(Zn) nearest neighbors is shorter in $CaMg_{2y}Zn_{2(1-y)}N_2$ compared to the distance between the Sr/Ca nearest neighbors in $Sr_xCa_{1-x}Zn_2N_2$ and the 11th nearest neighbor distance between the Mg/Zn sites is the same as the fifth nearest neighbor distance between the Sr/Ca sites in $CaZn_2N_2$. Three QSs are identified with the correlation function of the nearest neighbor pair cluster φ_{NN} close to the 800K value in Supplementary Fig. 19: one each at y = 0.25 and y = 0.75 with φ_{NN} = 0 and one at y = 0.5 with φ_{NN} = -1/3. The DFT and CE formation energies of these QSs as well as those of the PBE-GGA ground states are shown in Supplementary Fig. 20. The HSE06 band gaps of the QSs and end members are given in Fig. 4(b) in the main article.

$CaCd_{2z}Zn_{2(1-z)}N_2$. Finally, for the $CaCd_{2z}Zn_{2(1-z)}N_2$ system, DFT energies are calculated using a procedure similar to that used in $CaMg_{2y}Zn_{2(1-y)}N_2$. The DFT energies given in Supplementary Fig. 21(a) are very asymmetric, which is in sharp contrast to $Sr_xCa_{1-x}Zn_2N_2$ and $CaMg_{2y}Zn_{2(1-y)}N_2$. Supplementary Figure 21(c) shows the relaxed structure of the ground state at z = 0.75. The extensive relaxation of this structure means that the lattice model assumption that the cluster expansion formalism is based on cannot be applied to this system, hence phase diagram compilation through cluster expansion and Monte Carlo simulations is inappropriate.

Supplementary References

1 Momma, K. & Izumi, F. VESTA 3 for three-dimensional visualization of crystal, volumetric and morphology data. *J. Appl. Crystallogr.* **44**, 1272-1276 (2011).

2 Hatada, N. CHESTA. *http://www.aqua.mtl.kyoto-u.ac.jp/chestaEng.html* (2010).

3 Setyawan, W. & Curtarolo, S. High-throughput electronic band structure calculations: Challenges and tools. *Comput. Mater. Sci.* **49**, 299-312 (2010).

4 McQuarrie, D. A. & Simon, J. D. *Physical Chemistry: A Molecular Approach*. (University Science Books, 1997).

5 Kroll, P., Schröter, T. & Peters, M. Prediction of novel phases of tantalum(V) nitride and tungsten(VI) nitride that can be synthesized under high pressure and high temperature. *Angew. Chem. Int. Ed.* **44**, 4249-4254 (2005).

6 Chern, M. Y. & DiSalvo, F. J. Synthesis, structure, and properties of Ca_2ZnN_2. *J. Solid State Chem.* **88**, 528-533 (1990).

7 Zhang, X., Yu, L., Zakutayev, A. & Zunger, A. Sorting stable versus unstable hypothetical compounds: The case of multi-functional ABX Half-Heusler filled tetrahedral structures. *Adv. Funct. Mater.* **22**, 1425-1435 (2012).

8 Kuriyama, K., Kato, T. & Tanaka, T. Optical band gap of the filled tetrahedral semiconductor LiZnN. *Phys. Rev. B* **49**, 4511-4513 (1994).

9 Narang, P. *et al.* Bandgap tunability in $Zn(Sn,Ge)N_2$ semiconductor alloys. *Adv. Mater.* **26**, 1235-1241 (2014).

10 Fioretti, A. N. *et al.* Combinatorial insights into doping control and transport properties of zinc tin nitride. *Journal of Materials Chemistry C* **3**, 11017-11028 (2015).

11 Quayle, P. C., He, K., Shan, J. & Kash, K. Synthesis, lattice structure, and band gap of $ZnSnN_2$. *MRS Communications* **3**, 135-138 (2013).

12 Suda, T. & Kakishita, K. Band-gap energy and electron effective mass of polycrystalline Zn_3N_2. *J. Appl. Phys.* **99**, 076101 (2006).

13 Maruska, H. P. & Tietjen, J. J. The preparation and properties of vapor deposited single crystalline GaN. *Appl. Phys. Lett.* **15**, 327-329 (1969).

14 Walukiewicz, W. *et al.* Structure and electronic properties of InN and In-rich group III-nitride alloys. *J. Phys. D: Appl. Phys.* **39**, R83 (2006).

15 Reckeweg, O., Lind, C., Simon, A. & DiSalvo, F. J. Rietveld refinement of the crystal structure of α-Be_3N_2 and the experimental determination of optical band gaps for Mg_3N_2, Ca_3N_2 and $CaMg_2N_2$. *Z. Naturforsch. B* **58**, 159-162 (2003).

16 Shishkin, M., Marsman, M. & Kresse, G. Accurate quasiparticle spectra from self-consistent *GW* calculations with vertex corrections. *Phys. Rev. Lett.* **99**, 246403 (2007).
17 Lany, S. Band-structure calculations for the 3*d* transition metal oxides in *GW*. *Phys. Rev. B* **87**, 085112 (2013).
18 Kang, G., Kang, Y. & Han, S. Influence of wave-function updates in *GW* calculations on titanates. *Phys. Rev. B* **91**, 155141 (2015).
19 Grüneis, A., Kresse, G., Hinuma, Y. & Oba, F. Ionization potentials of solids: The importance of vertex corrections. *Phys. Rev. Lett.* **112**, 096401 (2014).
20 Bechstedt, F., Seino, K., Hahn, P. H. & Schmidt, W. G. Quasiparticle bands and optical spectra of highly ionic crystals: AlN and NaCl. *Phys. Rev. B* **72**, 245114 (2005).
21 Botti, S. & Marques, M. A. L. Strong renormalization of the electronic band gap due to lattice polarization in the *GW* formalism. *Phys. Rev. Lett.* **110**, 226404 (2013).
22 Bhandari, C., Lambrecht, W. R. L. & van Schilfgaarde, M. Quasiparticle self-consistent *GW* calculations of the electronic band structure of bulk and monolayer V_2O_5. *Phys. Rev. B* **91**, 125116 (2015).
23 Bergerhoff, G., Hundt, R., Sievers, R. & Brown, I. D. The inorganic crystal structure data base. *J. Chem. Inf. Comput. Sci.* **23**, 66-69 (1983).
24 Belsky, A., Hellenbrandt, M., Karen, V. L. & Luksch, P. New developments in the Inorganic Crystal Structure Database (ICSD): accessibility in support of materials research and design. *Acta Crystallogr. Sect. B* **58**, 364-369 (2002).
25 Kubaschewski, O. O., Alcock, C. B. & Spencer, P. J. *Materials thermochemistry*. 6 edn, (Pergamon Press, 1993).
26 Jain, A. *et al.* Commentary: The Materials Project: A materials genome approach to accelerating materials innovation. *APL Mater.* **1**, 011002 (2013).
27 Zhang, Y.-N. *et al.* Homogeneity range and crystal structure of the $Ca_2Mg_5Zn_{13}$ compound. *J. Alloys Compd.* **523**, 75-82 (2012).
28 Oganov, A. R. & Glass, C. W. Crystal structure prediction using *ab initio* evolutionary techniques: Principles and applications. *J. Chem. Phys.* **124**, 244704 (2006).
29 Togo, A., Oba, F. & Tanaka, I. First-principles calculations of the ferroelastic transition between rutile-type and $CaCl_2$-type SiO_2 at high pressures. *Phys. Rev. B* **78**, 134106 (2008).

30 Sanchez, J. M., Ducastelle, F. & Gratias, D. Generalized cluster description of multicomponent systems. *Physica A: Statistical Mechanics and its Applications* **128**, 334-350 (1984).

31 Ducastelle, F. *Order and Phase Stability in Alloys*. (North-Holland, 1991).

32 Fontaine, D. d. *Cluster approach to order-disorder transformations in alloys*, in *Solid State Phys.* Vol. 47, 33-176 (Academic Press, 1994).

33 Stone, M. Cross-validatory choice and assessment of statistical predictions. *J. Royal Statist. Soc. B* **36**, 111-147 (1974).

34 Van de Walle, A. & Ceder, G. Automating first-principles phase diagram calculations. *J. Phase Equilibria* **23**, 348 (2002).

35 Zunger, A., Wei, S. H., Ferreira, L. G. & Bernard, J. E. Special quasirandom structures. *Phys. Rev. Lett.* **65**, 353-356 (1990).

36 Seko, A. & Tanaka, I. Special quasirandom structure in heterovalent ionic systems. *Phys. Rev. B* **91**, 024106 (2015).
